# Differential Type I Interferon Signaling Is a Master Regulator of Susceptibility to Postinfluenza Bacterial Superinfection

**DOI:** 10.1128/mBio.00506-16

**Published:** 2016-05-03

**Authors:** Kelly M. Shepardson, Kyle Larson, Rachelle V. Morton, Justin R. Prigge, Edward E. Schmidt, Victor C. Huber, Agnieszka Rynda-Apple

**Affiliations:** a Department of Microbiology and Immunology, Montana State University, Bozeman, Montana, USA; b Division of Basic Biomedical Sciences, Sanford School of Medicine, University of South Dakota, Vermillion, South Dakota, USA

## Abstract

Bacterial superinfections are a primary cause of death during influenza pandemics and epidemics. Type I interferon (IFN) signaling contributes to increased susceptibility of mice to bacterial superinfection around day 7 post-influenza A virus (IAV) infection. Here we demonstrate that the reduced susceptibility to methicillin-resistant *Staphylococcus aureus* (MRSA) at day 3 post-IAV infection, which we previously reported was due to interleukin-13 (IL-13)/IFN-γ responses, is also dependent on type I IFN signaling and its subsequent requirement for protective IL-13 production. We found, through utilization of blocking antibodies, that reduced susceptibility to MRSA at day 3 post-IAV infection was IFN-β dependent, whereas the increased susceptibility at day 7 was IFN-α dependent. IFN-β signaling early in IAV infection was required for MRSA clearance, whereas IFN-α signaling late in infection was not, though it did mediate increased susceptibility to MRSA at that time. Type I IFN receptor (IFNAR) signaling in CD11c^+^ and Ly6G^+^ cells was required for the observed reduced susceptibility at day 3 post-IAV infection. Depletion of Ly6G^+^ cells in mice in which IFNAR signaling was either blocked or deleted indicated that Ly6G^+^ cells were responsible for the IFNAR signaling-dependent susceptibility to MRSA superinfection at day 7 post-IAV infection. Thus, during IAV infection, the temporal differences in type I IFN signaling increased bactericidal activity of both CD11c^+^ and Ly6G^+^ cells at day 3 and reduced effector function of Ly6G^+^ cells at day 7. The temporal differential outcomes induced by IFN-β (day 3) and IFN-α (day 7) signaling through the same IFNAR resulted in differential susceptibility to MRSA at 3 and 7 days post-IAV infection.

## IMPORTANCE

Approximately 114,000 hospitalizations and 40,000 annual deaths in the United States are associated with influenza A virus (IAV) infections. Frequently, these deaths are due to community-acquired Gram-positive bacterial species, many of which show increasing resistance to antibiotic therapy. Severe complications, including parapneumonic empyema and necrotizing pneumonia, can arise, depending on virulence factors expressed by either the virus or bacteria. Unfortunately, we are unable to control the expression of these virulence factors, making host responses a logical target for therapeutic interventions. Moreover, interactions between virus, host, and bacteria that exacerbate IAV-related morbidities and mortalities are largely unknown. Here, we show that type I interferon (IFN) expression can modulate susceptibility to methicillin-resistant *Staphylococcus aureus* (MRSA) infection, with IFN-β reducing host susceptibility to MRSA infection while IFN-α increases susceptibility. Our data indicate that treatments designed to augment IFN-β and/or inhibit IFN-α production around day 7 post-IAV infection could reduce susceptibility to deadly superinfections.

## INTRODUCTION

Despite medical advances, bacterial superinfections remain one of the primary causes of death during influenza A virus (IAV) pandemics, resulting in more-severe disease and mortality (1–3). Specifically, methicillin-resistant *Staphylococcus aureus* (MRSA) has been increasingly attributed to death from post-IAV bacterial superinfections (3–5). Bacterial superinfections mostly occur within the first 2 weeks of infection with IAV, with a peak increase in susceptibility occurring around day 7 postinfection (6, 7). We recently demonstrated that a delicate balance between IAV-induced interleukin-13 (IL-13) and gamma interferon (IFN-γ) affects susceptibility to superinfection (8, 9). However, the mechanism for regulation of this IL-13 response, and susceptibility to superinfection, remains unknown.

Type I IFNs play a well-characterized role in host responses against viruses and other intracellular pathogens. While frequently viewed as a common “type” of immune effector, type I IFNs are actually a large cytokine family including, among others, 14 IFN-αs and a single IFN-β (10). A product of multiple cell types, including macrophages, epithelial cells, and dendritic cells (11), most type I IFN responses are associated with the induction of proinflammatory genes and antiviral effectors mediated by signaling through the type I IFN receptor (IFNAR) (12). Induction of IFN-β precedes IFN-α chronologically, as it is produced after activation of cellular sensors, including RIG-I, MDA5, and Toll-like receptors (TLRs) (13). Production of IFN-β does not require signaling through IFNAR (14, 15); rather, binding of IFN-β to IFNAR induces subsequent transcriptional responses leading to IFN-α expression. That IFN-β can be produced early during the response, with IFN-α produced at a later time, suggests that this timing might have a role in disease outcome. The global type I IFN response is involved in both the control of viral spread and the expansion of lymphocytes to control intracellular infection (16–18). This demonstrates the dynamics of expression of type I IFNs and their contrasting signaling profiles, illustrating the importance of understanding their roles in different types of infections. This dichotomy in response is not uncommon within this pathway, as differential signaling in response to type I IFNs, depending on the ratio and expression of downstream mediators, particularly STATs, has been reported (19). Type I IFN signaling has also been shown to antagonize induction of IFN-γ, therein playing an immunomodulatory role (20). Here, we sought to determine the extent to which differential type I IFN signaling affects the lung, in particular as it relates to susceptibility to a postinfluenza bacterial superinfection.

The important contributions of the type I IFNs in viral defense were previously demonstrated in mice lacking either one of the subunits of the type I IFN receptor (*Ifnar1*^−/−^) or IFN-β are more susceptible to primary IAV infection (21–24). Additionally, wild-type (WT) mice that are infected with MRSA alone develop severe pneumonia, leading to death, while *Ifnar1*^−/−^ mice infected with MRSA have an improved outcome of infection (25–27). These disparate responses to single-pathogen infections reflect the complexity of the type I IFN signaling pathway and support the need to understand the role of individual type I IFNs during more complex infections, such as postinfluenza bacterial superinfection. In fact, the production of specific type I IFNs may directly contribute to the severity of these polymicrobial infections.

Previous work demonstrated that *Ifnar1^−/−^* mice were better able to contain a bacterial superinfection than WT mice during the clinical stage of IAV infection (7, 28–31), indicating that type I IFN signaling contributes to increased susceptibility to bacterial superinfection around day 7 post-IAV infection. Based on the differential outcomes of type I IFN signaling described above, we view type I IFNs as master regulators of susceptibility to superinfections. As such, we hypothesize that type I IFNs are acting upstream of IL-13 activation, and through their differential IFNAR-dependent signaling they are involved in altering the kinetics of IL-13/IFN-γ responses during the course of IAV infection. In the current study, we utilized a bacterial pneumonia model of postinfluenza superinfection to test this hypothesis and to determine the impact of type I IFNs in susceptibility to secondary bacterial pneumonia. To this end, we focused on the impact of IFN-α and IFN-β expression during both the early, preclinical stage of IAV infection (2 to 3 days post-IAV infection), where susceptibility to superinfection was reduced, and the late, clinical stage of IAV infection (7 days post-IAV infection), where mice were more susceptible to superinfection. Additionally, we also performed a comprehensive kinetic analysis of phenotypes of the immune cells in the lungs pre- and post-MRSA superinfection over the course of IAV infection to determine cell types that are involved in the response, such as neutrophils (CD11b^+^ CD11c^−^ Ly6G^+^ [32]), alveolar macrophages (CD11c^+^ CD11b^−^ SiglecF^+^ [33]), myeloid dendritic cells (CD11b^+^ CD11c_int_ major histocompatibility class II^+^ [MHC-II^+^] [33]), and inflammatory monocytes (CD11c^+^ CD11b^−^ SiglecF^−^ Ly6C^+^ [33]). Specifically, we sought to understand the role of the differential type I IFN production during postinfluenza bacterial superinfection by evaluating the host cellular responses that are affected by IFN-α or IFN-β signaling.

## RESULTS

### Mice deficient in type I IFN signaling are susceptible to MRSA superinfection early post-IAV infection.

Type I IFNs have been implicated in susceptibility to MRSA as either a single infection (25, 27) or as a bacterial superinfection, induced approximately 7 days post-IAV infection (7). Specifically, during superinfection at day 7 post-IAV infection, type I IFN signaling was shown to reduce the ability of mice to clear bacteria from the lungs (7). However, these cytokines are also important in early immune responses against IAV infection (21–23), creating a paradox in our understanding of the dynamics of host-pathogen interactions in polymicrobial infections. Additionally, we previously demonstrated that at day 3 post-IAV infection, WT mice had reduced susceptibility to superinfection (8). Therefore, we now asked whether and how type I IFN signaling was involved in the susceptibility of mice to bacterial superinfection early during IAV infection at day 3.

**FIG 1:**
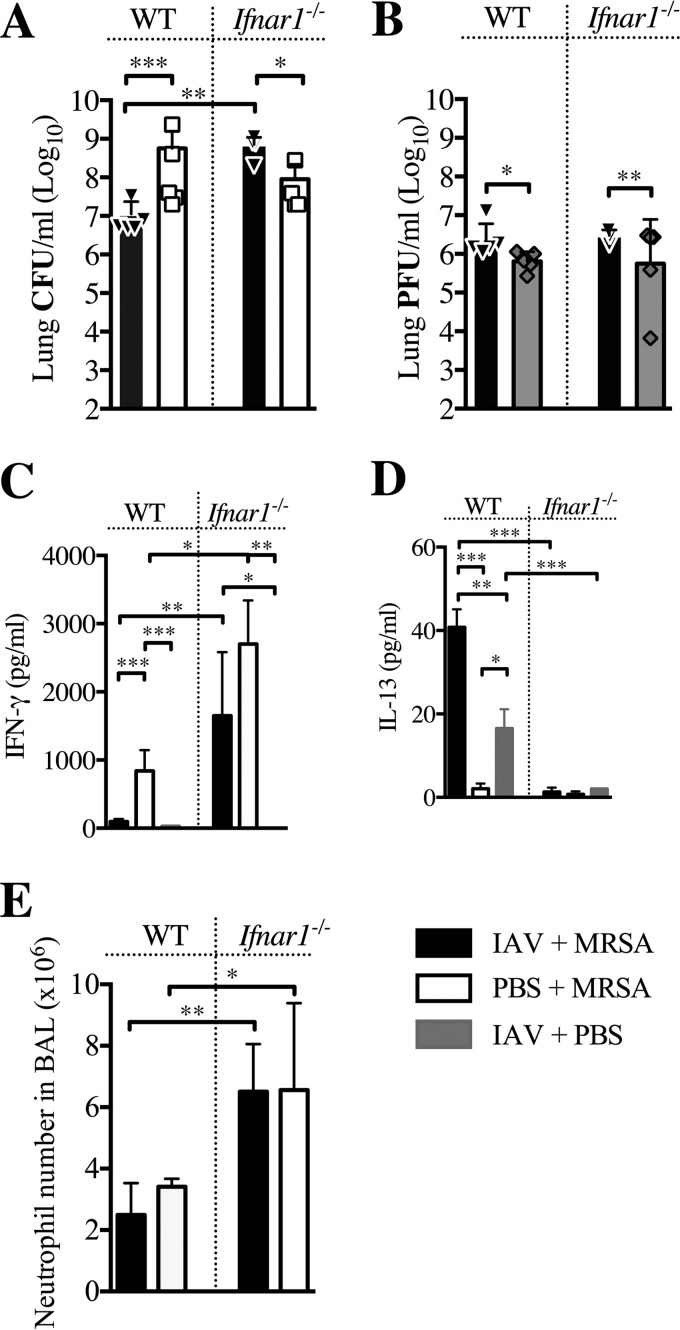
*Ifnar1* ^−/−^ mice are susceptible to secondary MRSA pneumonia early after IAV infection. *Ifnar1*^−/−^ and C57BL/6 (WT) mice were infected with IAV on day 0 and MRSA on day 3. (A and B) Bacterial (A) and viral (B) loads were evaluated 24 h after MRSA challenge (day 4 post-IAV infection). (C and D) Levels of IFN-γ (C) and IL-13 (D) were evaluated in cell-free BALF collected at the time of sacrifice. (E) Numbers of neutrophils in the BALF were determined at the time of sacrifice via differential counts. ***, *P* < 0.001; **, *P* < 0.01; *, *P* < 0.05.

Bacterial burdens in the lungs of *Ifnar1*^−/−^ mice superinfected with MRSA 3 days post-IAV infection were measured to determine whether the deficiency in signaling through IFNAR affects the early reduction in susceptibility to superinfection. Our previous work showed that when MRSA was delivered early post-IAV infection (day 3), the superinfected WT mice had reduced bacterial burdens compared to MRSA-only infected WT mice (8). However, *Ifnar1*^−/−^ mice (Fig. 1A), whether singly infected or superinfected, had increased numbers of bacteria in their lungs compared to superinfected WT mice (IAV plus MRSA), demonstrating that type I IFN signaling is required for reducing the susceptibility to superinfection at day 3 post-IAV infection. At 24 h post-MRSA challenge (day 4 post-IAV infection), both superinfected *Ifnar1*^−/−^ and WT mice had increases in viral burdens similar to those of their IAV-infected littermate controls, indicating that lack of type I IFN signaling does not affect host antiviral responses during the first 4 days of infection (Fig. 1B). This suggests that a host-derived mechanism controls the susceptibility, rather than virus-mediated mechanisms.

Previously, we demonstrated that mice with reduced susceptibility to superinfection with MRSA (at day 2 to 3 post-IAV infection) had elevated levels of IL-13 and reduced levels of IFN-γ in their lungs. In contrast, mice susceptible to superinfection (at day 7 post-IAV infection) had increased IFN-γ and no IL-13 in their bronchoalveolar lavage fluid (BALF) (8).

We determined that the increased susceptibility to MRSA superinfection early (day 3) after IAV infection of *Ifnar1*^−/−^ mice coincided with increased expression of IFN-γ and reduced levels of IL-13 in the BALF of these mice (Fig. 1C and D). This suggests a regulatory role for type I IFN signaling in susceptibility to superinfection and IFNAR-dependent IL-13 production during secondary bacterial superinfection. Interestingly, there was a 2-fold increase in neutrophils in the BALF of *Ifnar1*^−/−^ mice after bacterial infection, compared to WT mice, independent of whether mice had preceding IAV infection (Fig. 1E).

These results demonstrated that type I IFN signaling first reduces and then increases host susceptibility to superinfection between day 3 (Fig. 1A) and day 7 (7), respectively.

### Early post-IAV infection IFNAR signaling induces IL-13 production.

**FIG 2:**
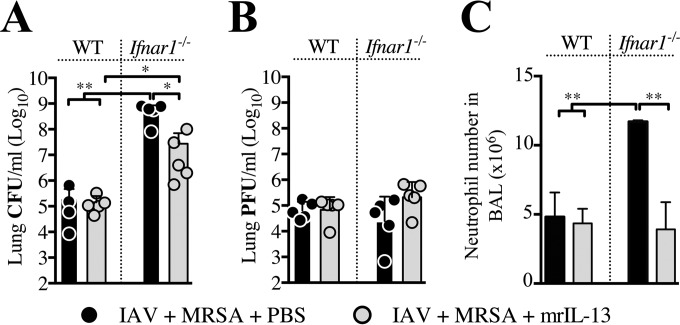
Lack of IL-13 production by superinfected *Ifnar1*^−/−^ mice contributes to the increased susceptibility to superinfection early after IAV infection. WT and *Ifnar1*^−/−^ mice were infected with IAV and treated either with 0.5 µg mrIL-13 or an equal volume of PBS at the time of challenge with MRSA on day 3 post-IAV infection, as well as 3 and 6 h later. Bacterial (A) and viral (B) loads as well as neutrophil numbers in the BALF (C) were evaluated 24 h after MRSA challenge (day 4 post-IAV infection). **, *P* < 0.01.

Absence of IL-13 in *Ifnar1*^−/−^ mice early post-IAV infection suggested that IFNAR-dependent signaling may act upstream of IL-13. To test this, we treated IAV-infected *Ifnar1*^−/−^ mice with exogenous IL-13 at the time of MRSA challenge (day 3). Within 24 h post-MRSA challenge, IL-13 treatment improved lung bacterial clearance compared to the response in phosphate-buffered saline (PBS)-treated superinfected *Ifnar1*^−/−^ mice (Fig. 2A). This suggested that the inability to produce IL-13 by *Ifnar1*^−/−^ mice is at least in part responsible for the increased susceptibility of these mice to superinfection at day 3 post-IAV infection. Addition of IL-13 to superinfected *Ifnar1*^−/−^ mice did not improve clearance of IAV from the lungs, but it reduced the level of neutrophils present in the BALF (Fig. 2B and C). Delivery of exogenous IL-13 to superinfected WT mice had no significant effect either on bacterial or viral burden or on neutrophil levels (Fig. 2A to C). However, in relation to MRSA-only infections of WT and *Ifnar1*^−/−^ mice, treatment with exogenous IL-13 of MRSA-only infected WT mice reduced bacterial burden within 24 h, but it did not affect the ability to clear MRSA from lungs of MRSA-only-infected *Ifnar1*^−/−^ mice (see Fig. S1A and B in the supplemental material). These results indicate that increased susceptibility to superinfection by deficiency of type I IFN-dependent signaling can be rescued by addition of IL-13 on day 3 post-IAV infection (8) and suggest that type I IFNs may be acting upstream of IL-13 production.

### IFNAR signaling between early (day 3) and late (day 7) IAV infection represents unique and independent events.

**FIG 3:**
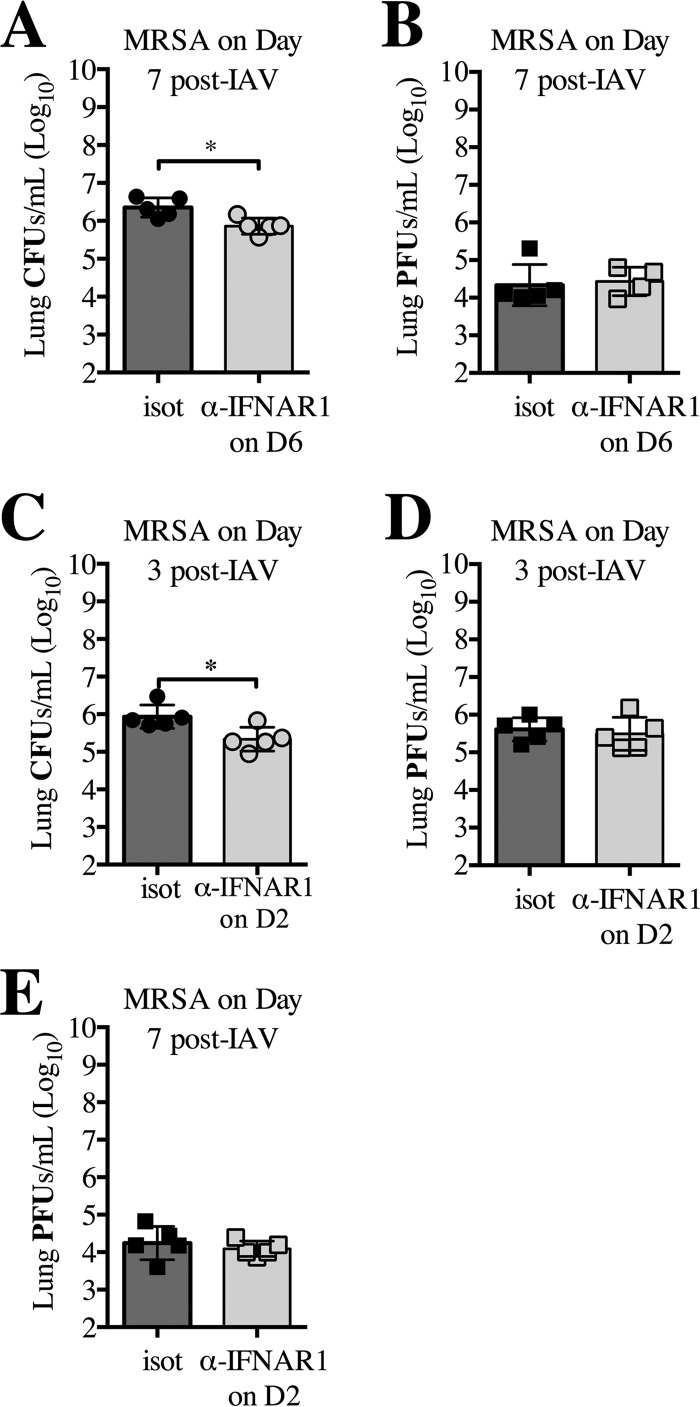
Inhibition of signaling through IFNAR1 at day 7 post-IAV infection reduces susceptibility to MRSA superinfection. WT mice were infected with IAV (day 0), treated with anti-IFNAR1 or isotype antibody on day 6 (D6), and challenged with MRSA on day 7. (A and B) Bacterial (A) and viral (B) loads were evaluated 24 h after MRSA challenge (day 8 post-IAV infection). (C to E) WT mice were infected with IAV on day 0, treated with anti-IFNAR1 antibody on day 2 (C, D, and E), and challenged with MRSA on day 3 (C and D). Bacterial (C) and viral (D) loads were evaluated 24 h after MRSA challenge (day 4 post-IAV infection). Viral load was measured in the lungs 7 days post-IAV infection (E). *, *P* < 0.05.

Our results demonstrated that type I IFN signaling responses and outcome of superinfection occurring both early (day 3) and late (day 7) during IAV infection are significantly different. Specifically, in contrast to day 3, superinfected *Ifnar1*^−/−^ mice cleared bacteria more efficiently than WT mice at day 7 post-IAV infection (7). To determine whether this contribution of type I IFN signaling to superinfection susceptibility at day 7 was a result of an inadequate initial response to IAV (occurring at day 3) or whether type I IFN signaling at the time of MRSA superinfection contributes to increased susceptibility, we used antibody treatment to block IFNAR1 24 h prior to MRSA challenge of WT mice at day 7 post-IAV infection. Inhibition of IFNAR1 signaling on day 6 post-IAV infection attenuated the susceptibility to superinfection at day 7 observed in isotype-treated superinfected WT mice (Fig. 3A), indicating that the type I IFN signaling later after IAV infection increases host susceptibility to superinfection at day 7. The inhibition of IFNAR1 on day 6 post-IAV infection did not affect the viral burden of the superinfected mice compared to isotype-treated superinfected mice (Fig. 3B), but it significantly reduced the extent of weight loss (see Fig. S2A in the supplemental material).

To understand the role of IFNAR signaling during the early response, we blocked IFNAR1 24 h prior to MRSA challenge at day 3. Interestingly, inhibition of type I IFN signaling reduced susceptibility to MRSA superinfection compared to isotype-treated superinfected WT mice (Fig. 3C). The inhibition of IFNAR1 on day 2 post-IAV infection did not affect viral burden (measured 24 h post-MRSA infection) in superinfected mice compared to the to isotype-treated superinfected WT mice (Fig. 3D), but it did significantly decrease weight loss (see Fig. S2B in the supplemental material). Inhibition of IFNAR1 did not affect the viral burden at day 7, when antibodies against this receptor were delivered at the apex of IAV infection (at day 2 post-IAV infection) (Fig. 3E).

These results demonstrate that the signaling of type I IFNs between days 3 and 7 post-IAV infection are both separate and distinct events that lead to differences in bacterial susceptibility. Additionally, the results up to this point indicate that while type I IFN signaling affects the host’s ability to clear bacteria during superinfection, it has minimal effects on the host’s ability to clear the virus at day 3 post-IAV infection, and it has no effect on viral clearance by day 7 post-IAV infection. Therefore, in the subsequent experiments presented here, we focused primarily on further understanding how type I IFN-dependent mechanisms lead to differences in susceptibility to the bacterial component of superinfection at days 3 and 7.

### Differential expression of IFN-α and IFN-β during the course of IAV infection contributes to the outcome of superinfection.

**FIG 4:**
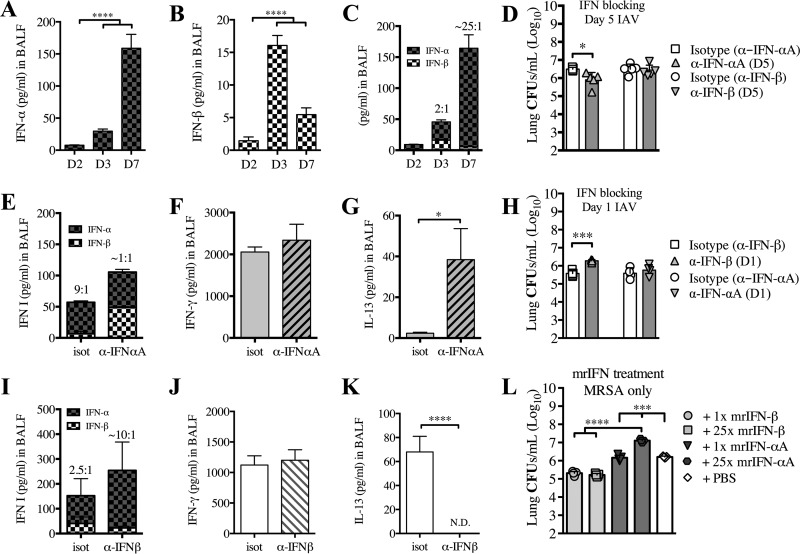
Reduction of IFN-β corresponds to increased susceptibility to post-IAV infection bacterial superinfection. WT mice were infected with IAV on day 0. Levels of IFN-α (A) and IFN-β (B) were evaluated in cell-free BALF collected at the time of sacrifice (days 2 [D2], 3, or 7 post-IAV challenge). (C) A combination of the results graphed in panels A and B, thus depicting ratios of IFN-α to IFN-β. (D to G) WT mice were infected with IAV on day 0, treated with anti-IFN-αA, anti-IFN-β, or isotype antibody on day 5, and challenged with MRSA on day 7. Bacterial burden (D) and the levels of IFN-α and IFN-β (E), IFN-γ (F), and IL-13 (G) were evaluated 24 h after MRSA challenge (day 8 post-IAV infection). (H to K) WT mice were infected with IAV on day 0, treated with anti-IFN-β, anti-IFN-αA, or isotype antibody on day 1, and challenged with MRSA on day 3. Bacterial burden (H) and the levels of IFN-α and IFN-β (I), IFN-γ (J), and IL-13 (K) were evaluated 24 h after MRSA challenge (day 4 post-IAV infection). (L) WT mice were treated with mrIFN-αA or mrIFN-β (1× dose was 10^4^ IU; 25× dose was 2.5 × 10^5^ IU) 24 h prior to being infected with MRSA. The bacterial burden was evaluated 24 h after MRSA challenge. Data shown are mean ± SEM results for 4 mice from one experiment. ****, *P* < 0.0001, ***, *P* < 0.001; *, *P* < 0.05.

Thus far, our results suggest that type I IFN signaling during the early and late response to IAV infection differentially contributes to susceptibility to superinfection. To determine whether IFN-α or IFN-β differently affect susceptibility to superinfection, we first examined the kinetics of IFN-α (consisting of all IFN-α subtypes) and IFN-β during the early (days 2 and 3) and late (day 7) post-IAV infection response. We found that IFN-α protein increased slightly in the BALF of mice at day 3 and peaked at day 7, whereas IFN-β peaked during the early stage of IAV infection at day 3 and was decreased at day 7 (Fig. 4A and B). Interestingly, we observed a progressive increase in the IFN-α:IFN-β ratio from day 3 (2:1) to day 7 (~25:1) (Fig. 4C).

Based on this observation, we sought to determine the impact of the production of IFN-α toward superinfection susceptibility by using an antibody to block IFN-α during the clinical stage of IAV infection. To minimize the complexity of blocking all IFN-αs, as there are 14 IFN-α subtypes, we decided to focus on understanding the role of IFN-αA, due to the extensive research available on IFN-αA’s role in enhancement of lymphocyte proliferation and apoptosis (34, 35). Inhibition of IFN-αA on day 5 post-IAV infection resulted in reduced susceptibility of mice to superinfection on day 7 compared to isotype-treated and superinfected WT mice (Fig. 4D). Importantly, reduced susceptibility of the IFN-αA-inhibited and superinfected mice correlated with a decrease in the IFN-α:IFN-β ratio and an increase in IL-13 production (Fig. 4E and G). Blocking IFN-αA did not affect the level of IFN-γ compared to isotype control mice (Fig. 4F), but it did significantly decrease the extent of weight loss (see Fig. S2C in the supplemental material). Importantly, inhibition of IFN-β at day 5 did not alter the susceptibility to post-IAV superinfection at day 7 (Fig. 4D).

We then determined whether the production of IFN-β plays a role in the early response, by using antibody to block IFN-β on day 1 post-IAV infection. Indeed, inhibition of IFN-β resulted in increased susceptibility of mice to superinfection on day 3 compared to isotype-treated superinfected WT mice (Fig. 4H), which corresponded with an increased IFN-α:IFN-β ratio and a decrease in IL-13 production (Fig. 4I and K). This effect was independent of IFN-γ, as blocking of IFN-β did not alter the level of IFN-γ compared to that in isotype-treated control mice (Fig. 4J), but it did significantly increase weight loss (see Fig. S2D in the supplemental material). Moreover, inhibition of IFN-αA at day 1 did not alter susceptibility to post-IAV superinfection at day 3 (Fig. 4H).

Considering the previous work that demonstrated a role for IFN-γ in post-IAV superinfection at day 7 (9), we wanted to determine the relationship between IFN-γ and type I IFNs during this response. We demonstrated that IFN-γ^−/−^ mice did not produce IFN-α following MRSA challenge on day 7 post-IAV infection, compared to the superinfected WT mice (see Fig. S3A in the supplemental material), further supporting our previously published results showing that IFN-γ^−/−^ mice were less susceptible than WT mice to superinfection at day 7 (8). Additionally, supplementing recombinant IFN-γ on day 5 post-IAV infection to *Ifnar1^−/−^* mice, which do not produce IFN-α, did not alter their susceptibility to MRSA superinfection at day 7 post-IAV infection (see Fig. S3B), further indicating that the role of IFN-γ during MRSA superinfection at day 7 post-IAV infection induces IFN-α production.

Additionally, we determined whether the type I IFNs had an effect on the susceptibility to MRSA infection alone by treating naive mice with either IFN-αA or IFN-β 24 h prior to MRSA infection. We found that treatment of naive mice with mouse recombinant IFN-β (mrIFN-β) reduced their susceptibility to MRSA infection compared with treatment of naive mice with either mrIFN-αA or PBS (Fig. 4L). Moreover, as IFN-β is known to have a much higher affinity for IFNAR (36–38), we decided to use a 25× dose of mrIFN-αA (based on the results shown in Fig. 4C) to determine whether at the higher concentration (and physiologically relevant ratio) IFN-αA is involved in susceptibility to MRSA. We found that the 25× dose of mrIFN-αA in naive mice increased their susceptibility to MRSA infection compared with naive mice treated with PBS or mrIFN-β (Fig. 4L). Interestingly, both the 1× and 25× doses of mrIFN-β also reduced susceptibility to MRSA (Fig. 4L), indicating that IFN-β in the lungs prior to MRSA infection induces a protective response, while IFN-αA induces a susceptible response.

Cumulatively, these results indicated that the reduced susceptibility to MRSA superinfection early (day 3) post-IAV infection is dependent on IFN-β signaling, whereas the increased susceptibility to superinfection late (day 7) post-IAV infection is mediated by IFN-α signaling in the absence of IFN-β signaling. Importantly, the protective IFN-β and nonprotective IFN-α responses can be manipulated at different times of susceptibility to alter the outcome.

### Type I IFN signaling in cells of bone marrow origin is necessary for early reduced susceptibility to superinfection.

**FIG 5:**
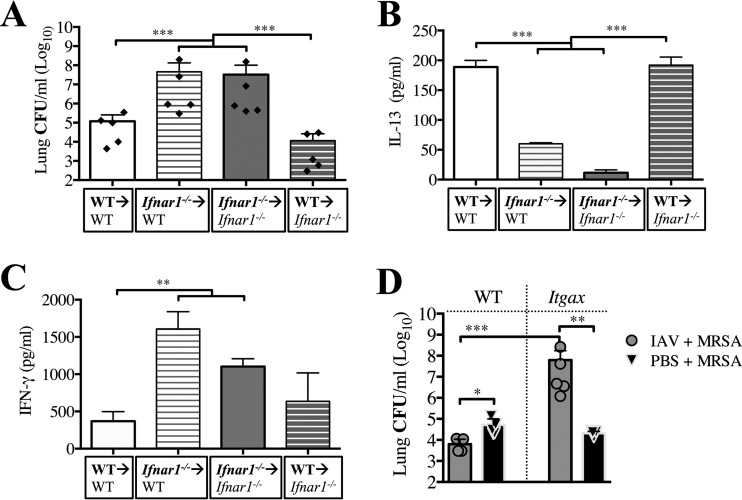
IFNAR signaling in BM-derived cells, specifically CD11c^+^ cells, is required for reduced susceptibility to superinfection early after IAV infection. BM chimeric mice were infected with IAV on day 0 and challenged with MRSA 3 days later (below each graph, the donor genotype is shown in bold, with the arrow indicating the recipient). (A) Lung bacterial burden was evaluated 24 h after MRSA challenge (day 4 post-IAV infection). (B and C) Levels of IL-13 (B) and IFN-γ (C) were evaluated in cell-free BALF collected at the time of sacrifice. (D) Itgax (*Ifnar1^fl/fl^*;*Itgax-cre*) and WT mice were infected with IAV (or inoculated with PBS) and challenged with MRSA (or PBS) 3 days later. Bacterial load was evaluated 24 h after MRSA challenge (day 4 post-IAV infection). ***, *P* < 0.001, **, *P* < 0.01, *, *P* < 0.05.

To identify cell types involved in type I IFN signaling and reduced susceptibility to superinfection early after IAV infection, we generated bone marrow (BM) chimeric mice that lack IFNAR signaling in either BM-derived or non-BM-derived cells. When superinfected with MRSA 3 days post-IAV infection, only the *Ifnar1^−/−^* chimeric mice that received WT BM transfer (**WT →** *Ifnar1^−/−^* mice [the bold font designates the genotype of the BM donor]) (Fig. 5A, gray slashed bar) showed reduced bacterial burden in their lungs to the same extent that control nonchimeric mice did (WT BM transferred to WT mice; **WT →** WT) (Fig. 5A, white bar). In contrast to **WT →** *Ifnar1^−/−^* mice, either the WT chimeric mice that received *Ifnar1^−/−^* BM transfer (***Ifnar1^−/−^*** → WT) or the nonchimeric ***Ifnar1^−/−^*** → *Ifnar1^−/−^* mice had almost 1,000-fold more bacteria in their lungs. This reduced susceptibility to superinfection 3 days post-IAV infection of **WT →** *Ifnar1^−/−^* chimeric mice and **WT →** WT nonchimeric mice coincided with significantly increased production of IL-13 in BALF and reduced production of IFN-γ (Fig. 5B and C). ***Ifnar1^−/−^*** → *Ifnar1^−/−^* nonchimeric mice and ***Ifnar1^−/−^*** → WT chimeric mice had substantially more bacteria in their lungs and significantly less IL-13 than did **WT →** WT nonchimeric mice, even though these ***Ifnar1^−/−^*** → *Ifnar1^−/−^* nonchimeric mice also had lower levels of IFN-γ than ***Ifnar1^−/−^*** → WT chimeric mice (Fig. 5B and C). No difference in viral burden was observed between the chimeric and nonchimeric mice (see Fig. S4A in the supplemental material). In summary, these results indicate that production of protective IL-13 during superinfection on day 3 requires IFNAR-dependent signaling in BM-derived cells.

To further discern which BM-derived cells require type I IFN signaling at day 3 post-IAV infection to reduce susceptibility to MRSA superinfection, we used mice that lacked IFNAR signaling exclusively in high CD11c^+^ cells (*Ifnar1^fl/fl^*;*Itgax-cre*). Compared to WT mice, *Ifnar1^fl/fl^*;*Itgax-cre* mice lost the ability to efficiently clear MRSA at day 3 post-IAV infection (Fig. 5D), suggesting that IFNAR signaling in high CD11c^+^ cells is necessary for the early reduction in susceptibility to MRSA superinfection. *Ifnar1^fl/fl^*;*Itgax-cre* mice infected with MRSA had only a minimal reduction in bacterial burden compared to the MRSA-only infected WT mice (Fig. 5D). Interestingly, superinfected *Ifnar1^fl/fl^*;*Itgax-cre* and WT mice, as well as mock-challenged IAV-infected WT mice, had similar viral burdens in their lungs 24 h postchallenge (day 4 post-IAV only) (see Fig. S4B in the supplemental material), suggesting that the lack of IFNAR signaling on high CD11c^+^ cells does not affect the IAV infection during the first 4 days. These results show that at day 3 post-IAV infection, type I IFN signaling in high CD11c^+^ cells protected mice from bacterial superinfection.

### IFNAR signaling in high CD11c^+^ cells is required for effectively killing MRSA at day 3, but not at day 7, post-IAV infection and corresponds with phenotypic changes of these cells during IAV infection.

**FIG 6:**
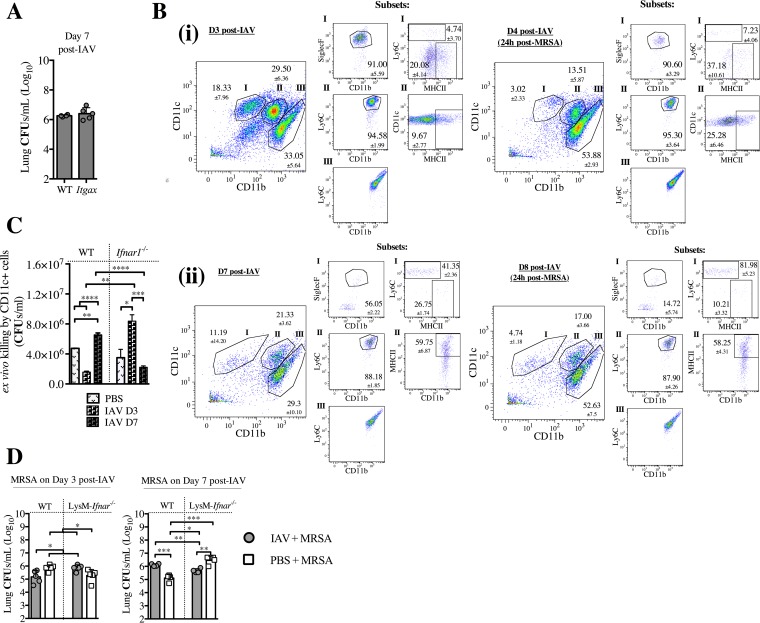
IFNAR signaling in CD11c^+^ cells is sufficient for killing MRSA at day 3 post-IAV infection, but not at day 7, and corresponds to phenotypic alterations occurring during IAV infection. (A) Itgax (*Ifnar1*^fl/*f*l^;*Itgax-cre*) mice were infected with IAV on day 0 and infected with MRSA on day 7. Bacterial burden was evaluated 24 h after MRSA challenge (day 8 post-IAV infection). (B) WT mice were sacrificed at the designated times post-IAV infection and post-MRSA infection (set i scatter plots, day 3 [D3] and day 4; set ii scatter plots, day 7 and day 8 post-IAV infection). Cells isolated from the BALF were stained and analyzed by using FACS. The gating for live cells was set on forward scatter versus side scatter. (i) In the left-most graph, staining for CD11c versus CD11b was determined by gating on the total live cells. The gating strategy entailed major gates (subsets) I (CD11c^+^ CD11b^−^), II (CD11b^+^ CD11c_int_), and III (CD11b^+^ CD11c^−^). The smaller groups of graphs for the subsets summarize scatter plots for SiglecF^+^, Ly6C^+^, and MHC-II^+^. Representative FACS plots with cell percentages (means and SEM) are shown. (C) WT and *Ifnar1*^−/−^ mice were infected with IAV on day 0 and CD11c^+^ cells were purified from the lungs at days 3 and 7, and their ability to kill MRSA following a 1.5-h incubation *ex vivo* was determined. (D) WT and LysM-Ifnar (*Ifnar1^fl/fl^*;*LysM^cre/cre^*) mice were infected with IAV on day 0 and infected with MRSA on either day 3 or day 7. The bacterial burden was evaluated 24 h after MRSA challenge (day 4 or 8 post-IAV infection). ****, *P* < 0.0001; ***, *P* < 0.001; **, *P* < 0.01; *, *P* < 0.05.

Due to the requirement of type I IFN signaling in high CD11c^+^ cells for reduced susceptibility of mice to superinfection at day 3 post-IAV infection, we sought to determine whether type I IFN signaling in CD11c^+^ cells was also responsible for the increased susceptibility to superinfection on day 7 post-IAV infection. We found that IFNAR signaling in high CD11c^+^ cells (alveolar macrophages [33]) was not involved in the IFNAR-dependent response at day 7 post-IAV, as the *Ifnar1^fl/fl^*;*Itgax-cre* mice (lacking IFNAR signaling in high CD11c^+^ cells) did not show a difference in susceptibility to post-IAV superinfection compared to superinfected WT mice (Fig. 6A). These results indicated that high CD11c^+^ cells were not involved in the type I IFN-mediated increase in susceptibility to superinfection at day 7 post-IAV infection.

To better understand what changes in the cellular environment of the lung accompany this type I IFN-dependent switch from reduced to increased susceptibility to post-IAV superinfection, we performed a comprehensive kinetic analysis of phenotypes of the immune cells in the lungs pre- and post-MRSA superinfection over the course of IAV infection. We found that, compared to BALF samples of mice infected with IAV for 3 days, there were fewer CD11c^+^ CD11b^−^ cells in BALF of mice infected with IAV for 7 days (Fig. 6B; see also Fig. S5 in the supplemental material). While about 91% of these CD11c^+^ CD11b^−^ cells at day 3 post-IAV infection were SiglecF^+^ (alveolar macrophages) (Fig. 6B, panel i), only about 55% were SiglecF^+^ at day 7 post-IAV infection (Fig. 6B, panel ii). Moreover, the CD11c^+^ CD11b^−^ cells at day 7 post-IAV infection (Fig. 6B, panel ii) were phenotypically distinct from that cell population at day 3 post-IAV infection (Fig. 6B, panel i) due to their expression of Ly6C (SiglecF^−^) and transition to inflammatory monocytes. Regardless of whether the MRSA challenge occurred on day 3 or 7 post-IAV infection, these SiglecF^+^ CD11c^+^ AMs were the main subpopulation of innate cells depleted, suggesting their importance in fighting MRSA superinfection (Fig. 6B; see also Fig. S5). Additionally, with the depletion of SiglecF^+^ CD11c^+^ cells after MRSA superinfection, there was also a significant increase in the level of CD11b^+^ CD11c^−^ cells (Fig. 6B) that were also Ly6G^+^ (neutrophils [32]) on days 4 and 8 post-IAV infection (24 h post-MRSA infection) (see Fig. S5), indicating their recruitment to the alveoli, consistent with involvement of these cells during MRSA infection (39).

In accordance with the change in phenotype and relative amount of CD11c^+^ cells between day 3 and day 7 post-IAV infection, we found that the CD11c^+^ cell population occurring at day 7 post-IAV infection consisted primarily of dendritic cells, monocytes, or alternatively activated macrophages (40) (Fig. 6B), but not the CD11c^+^ SiglecF^+^ cells found at day 3 post-IAV infection, are also important for killing MRSA. Specifically, CD11c^+^ cells harvested from day 7 post-IAV-infected WT mice were less able to effectively kill MRSA *ex vivo* than were CD11c^+^ cells from day 7 post-IAV-infected *Ifnar1*^−/−^ mice, but that CD11c^+^ cells from *Ifnar1*^−/−^ mice at day 7 post-IAV infection were equally effectively as CD11c^+^ cells from PBS-treated *Ifnar1*^−/−^ mice in killing MRSA (Fig. 6C). Consistent with the results with the *Ifnar1^fl/fl^*;*Itgax-cre* mice (Fig. 5D), CD11c^+^ cells from day 3 post-IAV-infected WT mice were more efficient at killing MRSA than CD11c^+^ cells from day 3 post-IAV-infected *Ifnar1*^−/−^ mice, supporting the involvement of IFNAR signaling in CD11c^+^ cells on day 3 post-IAV infection bacterial susceptibility. Cumulatively, these results indicate that type I IFN signaling in the different CD11c^+^ cell populations is involved in bacterial susceptibility at day 3 and 7 post-IAV infection. As there was not a significant reduction in MRSA killing by CD11c^+^ cells from day 7 post-IAV infection *Ifnar1*^−/−^ mice compared to CD11c^+^ cells from PBS-treated WT mice, our findings indicated that type I IFN signaling in cells other than CD11c^+^ cells was involved in the susceptibility to bacterial superinfection at day 7 post-IAV infection.

To determine which effector cells were involved in the immune defense at days 3 and 7 post-IAV infection, we sought to determine the susceptibility of mice lacking IFNAR signaling in myeloid cells (*Ifnar1^fl/fl^*;*LysM^cre/cre^*), as these cells are known to be involved in MRSA killing (41). We found that *Ifnar1^fl/fl^*;*LysM^cre/cre^* mice had increased susceptibility to bacterial superinfection at day 3 post-IAV infection compared to the superinfected WT mice (Fig. 6D), in agreement with the results with the *Ifnar1^fl/fl^*;*Itgax-cre* mice (Fig. 5D). Additionally, when examining day 7 post-IAV infection, we found that these *Ifnar1^fl/fl^*;*LysM^cre/cre^* mice had reduced susceptibility to superinfection compared to WT superinfected mice (Fig. 6D), indicating that type I IFN signaling specifically in myeloid cells other than CD11c^+^ cells increased host susceptibility to superinfection at day 7.

### IFNAR signaling impairs the ability of Ly6G^+^ cells to effectively kill MRSA during superinfection on day 7 post-IAV infection.

**FIG 7:**
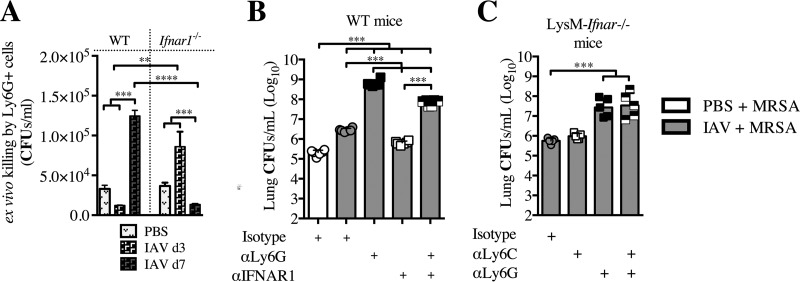
IFNAR signaling impairs Ly6G^+^ cell ability to kill MRSA on day 7 post-IAV infection. (A) WT and *Ifnar1*^−/−^ mice were infected with IAV on day 0, and Ly6G^+^ cells were purified from the lungs at day 3 and 7. The ability of these cells to kill MRSA following a 3-h incubation *ex vivo* was determined. (B) WT mice were infected with IAV on day 0 and treated with anti-IFNAR1 or isotype antibody on day 5.5 and/or anti-Ly6G or isotype antibody on day 6.5 and then infected with MRSA on day 7. Bacterial burden was evaluated 24 h after MRSA challenge (day 8 post-IAV infection). (C) LysM-*Ifnar* mice were infected with IAV on day 0, treated with antibody (anti-Ly6G, anti-Ly6C, or both) on day 6.5, and infected with MRSA on day 7. Bacterial burdens were evaluated 24 h after MRSA challenge (day 8 post-IAV infection). ***, *P* < 0.001.

Based on the observed increase in the Ly6G^+^ CD11b^+^ neutrophil cell population after MRSA superinfection (Fig. 6B), the reduced susceptibility of *Ifnar1^fl/fl^*;*LysM^cre/cre^* mice to superinfection on day 7 (Fig. 6D), and the fact that neutrophils are myeloid cells required for clearance of MRSA from the lung (41), we sought to determine whether IFNAR signaling in Ly6G^+^ cells contributes to either increased or reduced susceptibility of mice to superinfection. We found that Ly6G^+^ cells harvested from day 3 post-IAV-infected WT mice were able to effectively kill MRSA *ex vivo* and that this depended on whether they could signal through IFNAR, as Ly6G^+^ cells from *Ifnar1*^−/−^ mice at day 3 post-IAV infection were unable to effectively kill MRSA (Fig. 7A). Conversely, the Ly6G^+^ cells harvested from day 7 post-IAV-infected WT mice were not able to kill MRSA. However, if the day 7 post-IAV infection Ly6G^+^ cells were deficient in IFNAR signaling (from *Ifnar1*^−/−^ mice), they could kill as effectively as the day 3 post-IAV infection WT Ly6G^+^ cells. Based on the ability of day 7 post-IAV infection *Ifnar1*^−/−^ Ly6G^+^ cells to better kill MRSA, we evaluated their contribution to the reduced susceptibility to superinfection that occurs in *Ifnar1*^−/−^ mice (7) and WT mice in which IFNAR has been blocked (Fig. 3), at day 7 post-IAV infection. We found that depletion of Ly6G^+^ cells directly prior to superinfection on day 7 post-IAV infection of IFNAR1-blocked WT mice (αIFNAR + αLy6G) resulted in increased susceptibility to superinfection, contrasting with the reduced susceptibility found in the IFNAR1-blocked WT mice (Fig. 7B). The increased susceptibility of the αIFNAR/αLy6G-treated WT mice, however, was significantly less than the susceptibility of Ly6G-depleted mice. Additionally, the αIFNAR/αLy6G-treated WT mice compared to the αLy6G-treated WT mice had increased survival and less weight loss (see Fig. S6A and B in the supplemental material). These results indicate that at day 7 post-IAV infection, IFNAR signaling in Ly6G^+^ cells increases susceptibility to superinfection.

As discussed above, *Ifnar1^fl/fl^*;*LysM^cre/cre^* mice lack type I IFN signaling in granulocytes (Ly6G^+^ cells) and monocytes/macrophages (which in the lung consist mainly of CD11c^+^ macrophages/DCs and Ly6C^+^ monocytes). Thus, we next sought to determine whether type I IFN signaling in Ly6C^+^ cells contributes to susceptibility to superinfection. Depletion of Ly6C^+^ cells from superinfected *Ifnar1^fl/fl^*;*LysM^cre/cre^* mice did not significantly change susceptibility to MRSA, and it also did not augment the Ly6G^+^ cell depletion results (Fig. 7C; see also Fig. S7 in the supplemental material). In contrast, depletion of Ly6G^+^ cells from the superinfected *Ifnar1^fl/fl^*;*LysM^cre/cre^* mice resulted in increased susceptibility to MRSA and increased weight loss (Fig. 7C; see also Fig. S6C in the supplemental material). These results support a major role for IFNAR signaling in Ly6G^+^ cells, but not in Ly6C^+^ cells, at day 7 post-IAV infection. In summary, our data show that type I IFN signaling in high CD11c^+^ Ly6G^+^ and in Ly6G^+^ cells at day 3 and day 7 post-IAV infection, respectively, are involved in determining the susceptibility to bacterial superinfection.

## DISCUSSION

**FIG 8:**
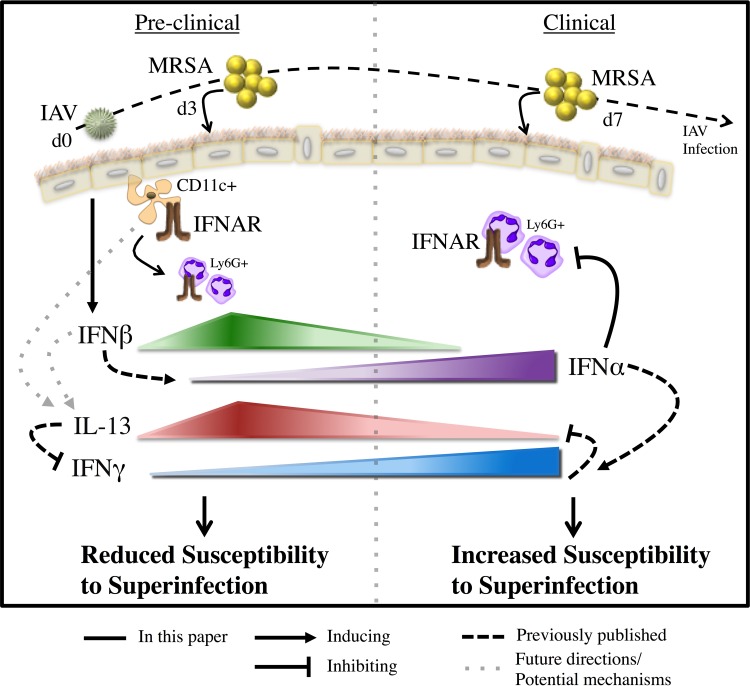
A model representation of the results and potential mechanisms presented, demonstrating the unique and distinct type I IFN signaling responses involved in post-IAV infection MRSA superinfection. We specifically determined that susceptibility to superinfection corresponded to the relative ratio of IFN-α and IFN-β. We found that increased IFN-β during the preclinical stage of IAV infection (day 3 [d3]) correlated with reduced susceptibility to MRSA superinfection, whereas increased IFN-α during the clinical stage of IAV infection (day 7) correlated with increased susceptibility to MRSA superinfection. We determined that type I IFN signaling in CD11c^+^ cells was required for the reduced susceptibility to superinfection during the preclinical stage (day 3) of IAV infection, but not during the clinical stage (day 7). We also found that type I IFN signaling in Ly6G^+^ cells was required for killing MRSA in the preclinical stage; however, type I IFN signaling in Ly6G^+^ cells during the clinical stage was detrimental for the control and susceptibility to superinfection. Further, we determined that this type I IFN-dependent signaling is involved in the IL-13/IFN-γ response known to be involved in susceptibility to superinfection (8). Future research will involve determining how type I IFN signaling regulates the production of IL-13.

Here, we have explored the mechanisms involved in type I IFN signaling, with emphasis on how the dynamic expression of IFN-β and IFN-α produced during early and late stages of IAV infection, respectively, contribute to bacterial superinfection susceptibility post-IAV infection (Fig. 8). We have demonstrated that the IFNAR-dependent signaling is involved in the IL-13/IFN-γ response, expanding upon the mechanisms involved in susceptibility to superinfection. Also, that type I IFN signaling affected different immune cells at either the preclinical (high CD11c^+^ and Ly6G^+^ cells) or the clinical (Ly6G^+^ cells) stages of IAV infection, corresponding with the levels of IFN-β and IFN-α, respectively, implicated differential type I IFN signaling as a major contributor to bacterial superinfection susceptibility.

Most cells within the host can produce type I IFNs, and their receptor, IFNAR, is broadly expressed on almost every cell (42). Previous work on understanding the cells responsible for the type I IFN response have focused mostly on single infections with either virus (IAV or respiratory syncytial virus [RSV]) or bacteria (MRSA or *Streptococcus pneumoniae*). During viral infection, intracellular receptors stimulate production of IFN-β from epithelial cells, independent of signaling through IFNAR (14, 42). This IFN-β then binds IFNAR, priming for the induction of IFN-αs through activation of additional transcription factors, which are produced mainly by plasmacytoid dendritic cells (pDCs) during IAV infection; interestingly, however, the absence of pDCs had no effect on IAV infection kinetics and disease progression (43, 44). As it relates to bacterial infections, much less is known about the contributions of type I IFN signaling during infection. However, what is known is that TLR signaling, as well as intracellular recognition of DNA during bacterial infection, activates IFN production. For example, different strains of *S. aureus* can activate different pattern recognition receptor (PRR) signaling cascades, specifically NOD2/IRF5 versus TLR9/IRF1, leading to different type I IFN responses that affect survival after infection (25, 27, 45). In this study, we sought to understand how the type I IFN signaling is involved in the immune response and mechanism for post-IAV infection bacterial superinfection.

Recently our group demonstrated that IL-13 was required for reducing bacterial susceptibility and that this IL-13 was produced by CD11c^+^ cells (46). In the current study, we expanded these findings by showing that IL-13 production during post-IAV infection bacterial superinfection requires IFNAR-dependent signaling in BM-derived cells and that IFNAR, specifically in high CD11c^+^ cells, was required to protect mice from increased bacterial burden at day 3 post-IAV infection MRSA superinfection (Fig. 8). Interestingly, the production of IL-13 required only BM-derived IFNAR-dependent signaling, indicating that the level of IL-13 induced by non-BM-derived cells was not sufficient to provide protection. This suggested that IFNAR-dependent signaling was required not only in BM-derived cells to protect from superinfection, but also for their subsequent production of IL-13. Based on our results, the CD11c^+^ cells responding to IFNAR-dependent signals and producing IL-13 are likely alveolar macrophages (high CD11c^+^ SiglecF^+^) and not myeloid DCs (CD11b^+^ CD11c_int_ MHC-II^+^) or pDCs, as DCs have low to intermediate CD11c and the peak of pDCs occurs around day 7 (44). A beneficial contribution of type I IFN production by AMs was recently demonstrated during RSV infection, supporting the results we report here with IAV (32). Our results demonstrate the importance of high CD11c^+^ cells in the early signaling response that is accountable for the reduced susceptibility to bacterial superinfection. Our results indicate that the CD11c^+^ cells that are important in the early signaling response are absent/diminished and have altered activation (CD11c^+^ Ly6c^+^ SiglecF^−^) later during infection and may consist of dendritic cells, monocytes, or alternatively activated macrophages (40).

Previously, the role of type I IFN signaling in susceptibility to bacterial superinfection with *Streptococcus pneumoniae* was examined by blocking IFNAR1 during the entirety of an IAV infection, and the outcome was a reduced susceptibility to superinfection (7). Essentially, this experiment, and experiments that use *Ifnar1*^−/−^ mice have demonstrated that inhibition of type I IFN signaling during the entire IAV infection reduces susceptibility to bacterial superinfection (29, 30). To expand upon these previous studies in many experiments in this study, we allowed the type I IFN responses to develop in response to the IAV infection and only blocked IFN signaling immediately prior to bacterial superinfection.

Our data show that the early type I IFN responses did not affect the reduced susceptibility that was found in the mice deficient in IFNAR at day 7, further supporting our position that a switch in the immune response occurs during the transition to the clinical stage of infection and that signaling from the type I IFNs is involved in this critical transition. These results complement and expand the findings of others (7, 29, 30), by showing this is not simply a result of defective antiviral responses. Rather, we demonstrate that type I IFN signaling directly influences the antibacterial response at the time of secondary bacterial infection, and these type I IFNs change the susceptibility to superinfection throughout the course of an IAV infection, allowing us to consider this ligand-receptor interaction as a regulator of this increased susceptibility.

Interestingly, when we examined the role of type I IFNs during the early response, we found that signaling directly following viral infection must control the effects of early IFNAR signaling. Due to IFN-β being constitutively expressed at low basal levels and not needing IFNAR signaling for priming of its own production (47), it is probable that the effects of the type I IFN signaling prior to IFNAR1 blocking may have provided the necessary events needed to more appropriately handle MRSA superinfection. These results support an early role for type I IFN signaling in establishing a lung environment that includes providing the necessary requirements to better control bacterial superinfection. We see a future therapeutic potential derived by both defining and exploiting this protective lung environment.

We determined that the differential type I IFN response was due to the early (day 3) and late (day 7) peaks of IFN-β and IFN-α, respectively, during the course of IAV infection. Additionally, the reduced ratio of IFN-α to IFN-β at day 3 and the increased ratio at day 7 suggest that the magnitude of IFNs expression, and not just their presence, may determine the susceptibility to superinfection. These results did not appear to depend on IFN-γ, as the level of IFN-γ was not affected by antibody depletion of either IFN-αA or IFN-β. However, our results implicate IFN-γ as at least partially responsible for the induction of IFN-α, indicating that type II IFNs may not be the only contributor to superinfection susceptibility. However, antibody depletion of either IFN-αA or IFN-β did result in the increase and decrease of IL-13 production, respectively, suggesting that, whereas the protective IL-13 response during superinfection is dependent on IFNAR signaling, the IL-13 production may not be directly induced by type I IFNs alone (data not shown), the differential signaling via IFNAR is further supported by the ability to achieve similar reduced susceptibility and signaling responses by blocking at least one of the IFN-αs at day 7, while other IFN-αs were still detectable and present.

Together, our data provide support for the level, timing, and ratio of type I IFNs in a mechanism associated with susceptibility to bacterial superinfection over the course of IAV infection. Interestingly, IFN-β has a demonstrated benefit for the host response against IAV, as mice that are deficient in IFN-β are more susceptible to virus infection (21, 24). Alternatively, in the case of MRSA infection, IFN-β induced following infection was detrimental to the survival of the mice (25, 27). This difference in the role of IFN-β toward susceptibility to MRSA is probably due to the timing of the IFN-β response, as we determined that IFN-β administered prior to MRSA infection was beneficial, while others have determined that IFN-β administration after MRSA infection worsened the infection outcome (25, 27). Moreover, we determined that a physiologically relevant dose of IFN-αA administered prior to MRSA infection increased susceptibility to infection. There is much evidence that depicts differences in the function of type I IFNs (36, 48, 49), suggesting that there is specificity in the signaling pathways activated by the different type I IFNs, which may be related to their levels of expression. Specifically, it was demonstrated that type I IFNs can activate the “robust” and “tunable” genes differently or to the same extent, but without the same degree of antiviral activity, based on their level and the cell type (50). Additionally, type I IFNs have also been shown to suppress the immune system, allowing viral persistence with viruses such as lymphocytic choriomeningitis virus, further supporting that differential signaling affects the immune response and the outcome of infection (51, 52). Additionally, IFN-β was recently found to form a complex with IFNAR1 in the absence of the heterodimeric receptor’s partner IFNAR2, suggesting that differences in downstream signaling by the individual type I IFNs is selective and may differ based on receptor binding conditions (48, 53). Potentially, these receptor interactions, and the downstream signals they initiate, may be affected by the levels of the individual type I IFNs.

One condition of IAV infection that is thought to aid in the susceptibility during the clinical stage is the changing of the pulmonary environment. IAV infection results in exhaustion of neutrophils and macrophages, with downregulation of innate receptors, attenuation of antimicrobial peptide production, downregulation of Th17 responses, and IL-1β production, but it also disrupts and damages the respiratory epithelium (29, 54–56). These events follow the general timeline of susceptibility to bacterial superinfection that we have demonstrated and suggest that changes in the cellular components of the pulmonary environment influence this response. Consistent with this change, we found that IFNAR signaling in Ly6G^+^ cells contributes to increased susceptibility to superinfection during the clinical stage of IAV infection (day 7) (Fig. 8). The role of Ly6G^+^ cells was previously demonstrated to be involved in the response to *S. pneumoniae* superinfection during the clinical stage of IAV infection (9, 56). Specifically, a reduced ability of Ly6G^+^ cells from IAV-infected mice to induce reactive oxygen species was found to contribute to this increase in susceptibility (41). It will be important to determine how IAV-induced type I IFN signaling affects the ability of Ly6G^+^ cells to protect against superinfection, specifically, their antibacterial defense mechanisms, and the role of IFN-β in this response. In this regard, we found that at the early stage (day 3) of IAV infection, *Ifnar1*^−/−^ mice recruited more neutrophils than their WT counterparts, whereas other researchers have found decreased neutrophil recruitment during the day 7 response in superinfected *Ifnar1*^−/−^ mice (29). Thus, it is possible that an overabundant inflammatory cell response caused by the increased cell recruitment and/or lack of an appropriate type I IFN signaling response in the cells could contribute to this susceptibility to superinfection in *Ifnar1*^−/−^ mice at day 3 post-IAV infection.

The signaling pathways involved in type I IFN responses involve multiple transcription factors, including STATs and IRFs, and this complexity depends on the cell type responding to the signal and the subsequent activation of downstream effectors (11). Here, we demonstrate the requirement of IFNAR-dependent signaling in the induction of IL-13, which mediates the host response to effectively kill bacteria during post-IAV infection superinfection (Fig. 8). There is evidence that type I IFNs induce IL-13 production in certain host cells through STAT2 and STAT6 complexes (57). Importantly, the production of IL-13 is controlled by the transcription factor STAT6, and through autocrine signaling, via the IL-13 receptor, the levels of IL-13 increase further. Recently, the adaptor protein endoplasmic reticulum IFN stimulator (STING, also known as MITA/ERIS), along with MAVS and TBK1, were identified for their contribution to STAT6 activation during viral infection with Semiliki Forest virus and herpes simplex virus 1 (HSV-1) (58). That study further defined STAT6 as a critical factor for survival during primary viral infection. STING was found to have a critical role for production of type I IFNs that directly influenced survival after HSV-1 infection (59). The observation that STAT6/STING contribute to type I IFN production and survival during primary virus infection suggests that STING and STAT6 may be involved in the control of IL-13 and type I IFN production and susceptibility to post-IAV infection superinfection. A future understanding of the immune pathways that are activated during the different stages of IAV infection will allow us to determine the mechanism through which type I IFN-dependent signaling is involved in the induction of IL-13 and in protection against superinfection.

The results presented here provide new insights into a distinct role for type I IFNs in controlling susceptibility to post-IAV infection superinfection. Our data demonstrate that a unique type I IFN signaling pattern occurs in distinct cell types throughout IAV infection (Fig. 8). As such, we determined that type I IFN signaling through both high CD11c^+^ and Ly6G^+^ cells at day 3 was beneficial for superinfection outcome, whereas signaling through Ly6G^+^ cells at day 7 was detrimental. Importantly, we have provided direct evidence through the utilization of blocking antibodies that IFN-β contributes to reduced susceptibility at day 3 post-IAV infection, and IFN-α plays a key role in the increased susceptibility at day 7 post-IAV infection, supporting a mechanism involving precisely controlled and differential type I IFN signaling. These data reflect the importance of using the knowledge of single infections to determine the potential outcomes of complex polymicrobial infections. Using this approach, our goal is to better understand how to treat patients with primary IAV infections, so that the viral infection does do not progress into deadly superinfection.

## MATERIALS AND METHODS

### Mice.

C57BL/6J mice, B6.Cg-Tg(Itgax-cre)1-1Reiz/J mice, and B6.129P2-Lyz2^*tm1*(*cre*)*Ifo*^*/*J mice (“*LysM^cre^*”) were obtained from Jackson Laboratories (Bar Harbor, ME). A conditional *null* allele of the *Ifnar1* gene (chromosome 16), entitled *Ifnar1^fl^* for “floxed,” in which exon 3 is flanked by *Lox*P sites, was designed, produced on a purely C57BL/6 background, and validated at Montana State University (MSU); a complete description of this allele, including a detailed analysis of its activity, is described in reference 60. The *Ifnar1^fl^* mouse line will be available from the Jackson Laboratories as stock 028256. B6.SJL-*PtprcaPepc^b^*/BoyCrCrl mice were obtained from Charles River Laboratories. To generate a full-body *Ifnar1^−/−^* colony on a purely C57BL/6 background, *Ifnar1^fl/fl^* mice were bred across the X-linked Deleter-cre allele (61) on a highly back-crossed C57BL/6 background, pups bearing the recombined *Ifnar1^−/−^* (null) allele were selected, and these were bred to homozygosity and away from the Deleter-cre transgene. For tissue-specific disruption of *Ifnar1*, colonies were generated that were *Ifnar1^fl/fl^*;*Itgax-cre^1^* (homozygous for the *Ifnar1^fl^* allele and carrying one copy of the *Itgax-cre* transgene) or *Ifnar1^fl/fl^*;*LysM^cre/cre^* (homozygous for both the *Ifnar1^fl^* allele on chromosome 16 and the *LysM^cre^* knock-in allele on chromosome 10). All mice used in this study were between 7 and 8 weeks of age unless specifically indicated.

BM chimeric mice were generated by reconstituting irradiated (1,000 cGy) recipient mice (CD45.1^+^ C57BL/6 or *Ifnar1^−/−^*) with 10^7^ *Ifnar1^−/−^* or CD45.1^+^ C57BL/6 BM cells. Following transplant, mice were treated with trimethoprim-sulfamethoxazole in the drinking water for 14 days and rested for 6 to 8 weeks prior to use in experiments (8).

Mice were maintained at the Montana State University (MSU) Animal Resources Center. Mice were weighed and monitored for signs of morbidity and mortality. All care and procedures were in accordance with the recommendations of the National Institutes of Health, the U.S. Department of Agriculture, and the *Guide for the Care and Use of Laboratory Animals* (8th ed.) (62). Animal protocols were reviewed and approved by the MSU Institutional Animal Care and Use Committee (IACUC), protocol 2014-16 and the MSU federal-wide assurance for animal welfare (A3627-01). MSU is accredited by the Association for Assessment and Accreditation of Laboratory Animal Care (AAALAC; number 713).

### Infections and bacterial and viral burdens.

For nonsurgical intratracheal (i.t.) inoculations, mice were lightly anesthetized as described elsewhere (63, 64) and instilled with 1,500 PFU of mouse-adapted IAV strain A/PR8/8/34 (PR8; H1N1) in 100 µl of normal saline. The LAC strain of MRSA (pulsed-field type USA300) was a kind gift from Jovanka Voyich at MSU. Doses of 10^8^ CFU were used for infection; however, between experiments there was inoculum-to-inoculum variability. Our previously described procedures for determining CFU (46) and PFU (65) were followed. CFU and PFU were determined from the lung homogenate samples.

### Preparation of BALF samples and cytokine analyses.

Mice were sacrificed by intraperitoneal (i.p.) administration of 90 mg/kg of body weight sodium pentobarbital followed by blood collection. BALF was obtained by lavaging the lungs with 2 ml of 3 mM EDTA in PBS (8). Levels of IFN-α (31.3 to 20,00 pg/ml), IL-13 (4 to 500 pg/ml), IFN-β (1.9 to 500 pg/ml), and IFN-γ (15.6 to 1,000 pg/ml) in cell-free BALF were determined by using a sandwich enzyme-linked immunosorbent assay kit with the BALF samples (Ready-SET-Go; eBioscience, San Diego, CA, or BioLegend). Results from analysis of BALF samples are reported as means ± standard errors of the means (SEM) from 5 mice (tested in triplicate).

### Flow cytometry.

A single-cell suspension was obtained by centrifugation of BALF specimens. Red blood cells were lysed with AKC lysis buffer (150 mmol/liter NH_4_Cl, 1 mmol/liter KHCO_3_, and 0.1 mmol/liter Na_2_-EDTA; pH 7.2 to 7.4) at room temperature for 5 min. Cells were washed with PBS twice and blocked with Fc receptor block (2.4G2 hybridoma, made in house) for 20 min at room temperature. Cells were stained with: CD11c (clone N418; BioLegend), CD11b (clone M1/70; eBioscience), Ly6G (clone 1A8; BioLegend), MHC-II (clone M5/114.15.2; BioLegend), SiglecF (clone E50-2440; BD Bioscience), and Ly6C (clone HK1.4; BioLegend). After a 30-min incubation, cells were washed twice in fluorescence-activated cell sorting (FACS) buffer (1% fetal bovine serum–PBS). Samples were acquired on a FACSCanto apparatus running FACSDiva software (both obtained from BD Bioscience). Approximately 50,000 events were routinely acquired per sample. FlowJo software (Tree Star, Inc., Ashland, OR) was used for analysis.

### Treatment of mice with recombinant proteins and antibodies.

Mice received the indicated doses of mrIL-13 (0.5 µg/dose; BioLegend), mrIFN-γ (1.5 µg/dose; BioLegend), or 10^4^ or 2.5 × 10^5^ IUs of mrIFN-β or mrIFN-αA (PBL Assay Science, Piscataway, NJ) by i.t instillation. Antibody depletions were performed as stated for each experiment. For anti-IFNAR1 blocking treatment (7, 66), we used the following antibodies: 1.5 mg of anti-IFNAR1 (MAR1-5A3) monoclonal antibody (MAb) or IgG1 isotype control antibody (Leinco Technologies, St. Louis, MO) administered i.p. For IFN-α/IFN-β treatment (67), we used 0.25 mg of anti-IFN-α (RMMA-1) MAb/anti-IFN-β (RMMB-1) MAb or an IgG1 isotype control antibody (~95 and ~90% efficacy, respectively; PBL Assay Science, Piscataway, NJ) administered i.p. For anti-IFNAR1/Ly6G treatment, we used 1.75 mg of anti-IFNAR1 (MAR1-5A3) MAb or IgG1 isotype control antibody (Leinco Technologies; St. Louis, MO) administered i.p. and 0.25 mg of anti-Ly6G (1A8; average ~90% depletion) MAb or IgG2a isotype control antibody (Leinco Technologies, St. Louis, MO) administered i.t. For anti-Ly6C/Ly6G depletion treatment, we used 0.25 mg anti-Ly6G (1A8; average ~97% depletion) or 0.25 mg anti-Ly6C (both from Bio X Cell, West Lebanon, NH) MAb or IgG2a isotype control antibody (Leinco Technologies, St. Louis, MO) administered i.t. (see Fig. S7 in the supplemental material).

### Bacterial killing assays.

A single-cell suspension obtained after red blood cell lysis and filtration (50-µm pore size) of lung homogenate (Ly6G) or collagenase-digested lung homogenate (CD11c) was run through magnetic-activated cell sorting (MACS)-positive selection columns to obtain a pure Ly6G^+^ or CD11c^+^ cell population (~97% purity). The purity of all fractions was confirmed by following the manufacturers’ recommendations (Miltenyi Biotec, CA). Our previously described procedures for the bacterial killing assay (46) were followed.

### Statistical analyses.

Unless otherwise specified in the figure legends, reported results are means ± standard deviations (SD) of 5 mice/group from a single experiment. Each experiment for which results are presented in the manuscript was independently performed at least twice with similar results. The differences between treatment groups were analyzed using Student’s *t* test or analysis of variance. For the differences in survival rates, Kaplan-Meier curves were plotted and analyzed using Prism software (version 4.0; GraphPad, La Jolla, CA). Statistical differences with *P* values of <0.05 were considered significant.

## SUPPLEMENTAL MATERIAL

Figure S1  (A) C57BL/6 (WT) mice were infected with MRSA (3 × 10^8^ CFU). At the time of MRSA challenge and at 3 and 6 h after MRSA infection, mice received i.t. instillations of mrIL-13 (1 µg/dose). (B) *Ifnar1*^−/−^ mice were infected with MRSA (1 × 10^8^ CFU). At the time of MRSA challenge and at 3 and 6 h after MRSA infection, mice received i.t. instillations of mrIL-13 (0.5 µg/dose). Lung bacterial burdens were evaluated at 24 h after MRSA infection. ****, *P* < 0.0001. Download Figure S1, TIF file, 0.5 MB

Figure S2  Body weight data from the experiments shown in Fig. 3. (A) WT mice were infected with IAV (day 0), treated with anti-IFNAR1 antibody on day 6, and challenged with MRSA on day 7. (B) WT mice were infected with IAV on day 0, treated with anti-IFNAR1 antibody on day 2, and challenged with MRSA on day 3. (C) WT mice were infected with IAV on day 0, treated with anti-IFN-αA or isotype antibody on day 5, and challenged with MRSA on day 7. (D) WT mice were infected with IAV on day 0, treated with anti-IFN-β or isotype antibody on day 1, and challenged with MRSA on day 3. **, *P* < 0.01; *, *P* < 0.05. Download Figure S2, TIF file, 1.2 MB

Figure S3  (A) IFN-γ^−/−^ and C57BL/6 (WT) mice were infected with IAV on day 0 and challenged with MRSA on day 7. The levels of IFN-γ were evaluated in cell-free BALF collected at the time of sacrifice (day 8 post-IAV infection). (B) *Ifnar1*^−/−^ mice were infected with IAV on day 0, treated with 1.5 µg mrIFN-γ on day 5, and challenged with MRSA on day 7. The lung bacterial burden was evaluated at 24 h after MRSA infection (day 8 post-IAV infection). **, *P* < 0.01. Download Figure S3, TIF file, 0.2 MB

Figure S4  BM chimeric mice were infected with IAV on day 0 and challenged with MRSA 3 days later (the donor genotype is shown in bold, with an arrow indicating the recipient). (A) Viral burden was measured in the lungs 24 h after MRSA challenge (day 4 of IAV infection). Itgax (*Ifnar1^fl/fl^*;*Itgax-cre*) and WT mice were infected with IAV (or inoculated with PBS) and challenged with MRSA (or PBS) 3 days later. (F) The viral load was evaluated 24 h after MRSA challenge (day 4 post-IAV infection). Download Figure S4, TIF file, 0.5 MB

Figure S5  WT mice were sacrificed at the designated times post-IAV infection and post-MRSA infection (day 3 [d3], d4, and d7 or d8 post-IAV infection). Cells isolated from the BALF were stained and analyzed by FACS. The live cells gate was set on forward scatter (FCS) versus side scatter (SSC). Staining for CD11c versus CD11b was determined by gating total live cells. The gating strategy was as follows: for station 1 (A and C), major gates I (CD11c^+^ CD11b^−^), II (CD11b^+^ CD11c_int_), and III (CD11b^+^ CD11c^−^); subset gates I (SiglecF^+^, Ly6C^+^, and MHC-II^+^), II (Ly6C^+^ and MHC-II^+^); III (Ly6C^−^). For station 2, subset gates were as follows: I (Ly6C^+^ and MHC-II^+^), II (Ly6C^+^ and MHC-II^+^), and III (Ly6G^+^). The number of whole cells for major and subset gates are displayed. *, *P* < 0.05; ψ combined with *, the *P* value is for comparison of that cell type at day 3 and at day 7. Download Figure S5, TIF file, 2.6 MB

Figure S6  Body weight and survival data from Fig. 3 experiments. (A and B; extending data presented in Fig. 7B) WT mice were infected with IAV on day 0, treated with anti-IFNAR1 antibody on day 5.5 and/or anti-Ly6G antibody on day 6.5, and then infected with MRSA on day 7. (C; extending data presented in Fig. 7C) LysM-*Ifnar* mice were infected with IAV on day 0, treated with antibody (anti-Ly6G, anti-Ly6C, or both) on day 6.5, and infected with MRSA on day 7. Download Figure S6, TIF file, 0.7 MB

Figure S7  Cellular depletion plots for the data presented in Fig. 7B (A) and Fig. 7C (B). Cells isolated from the BALF were stained and analyzed by FACS. The live cells gate was set for forward scatter (FCS) versus side scatter (SSC). Staining for CD11c versus CD11b was determined by gating on total live cells. Plots shown are CD11b^+^ cells stained for Ly6G and Ly6C. (A) WT mice were infected with IAV on day 0, treated with anti-IFNAR1 antibody on day 5.5 and/or anti-Ly6G antibody on day 6.5, and then infected with MRSA on day 7. (B) LysM-*Ifnar* mice were infected with IAV on day 0, treated with antibody (anti-Ly6G, anti-Ly6C, or both) on day 6.5, and infected with MRSA on day 7. Download Figure S7, TIF file, 1.9 MB
