# Supplementary figures and images for: Differential Type I Interferon Signaling Is a Master Regulator of Susceptibility to Postinfluenza Bacterial Superinfection
Source: mBio. 2016 May 3;7(3):e00506-16. doi: 10.1128/mBio.00506-16 (PMC4959663; doi:10.1128/mBio.00506-16)

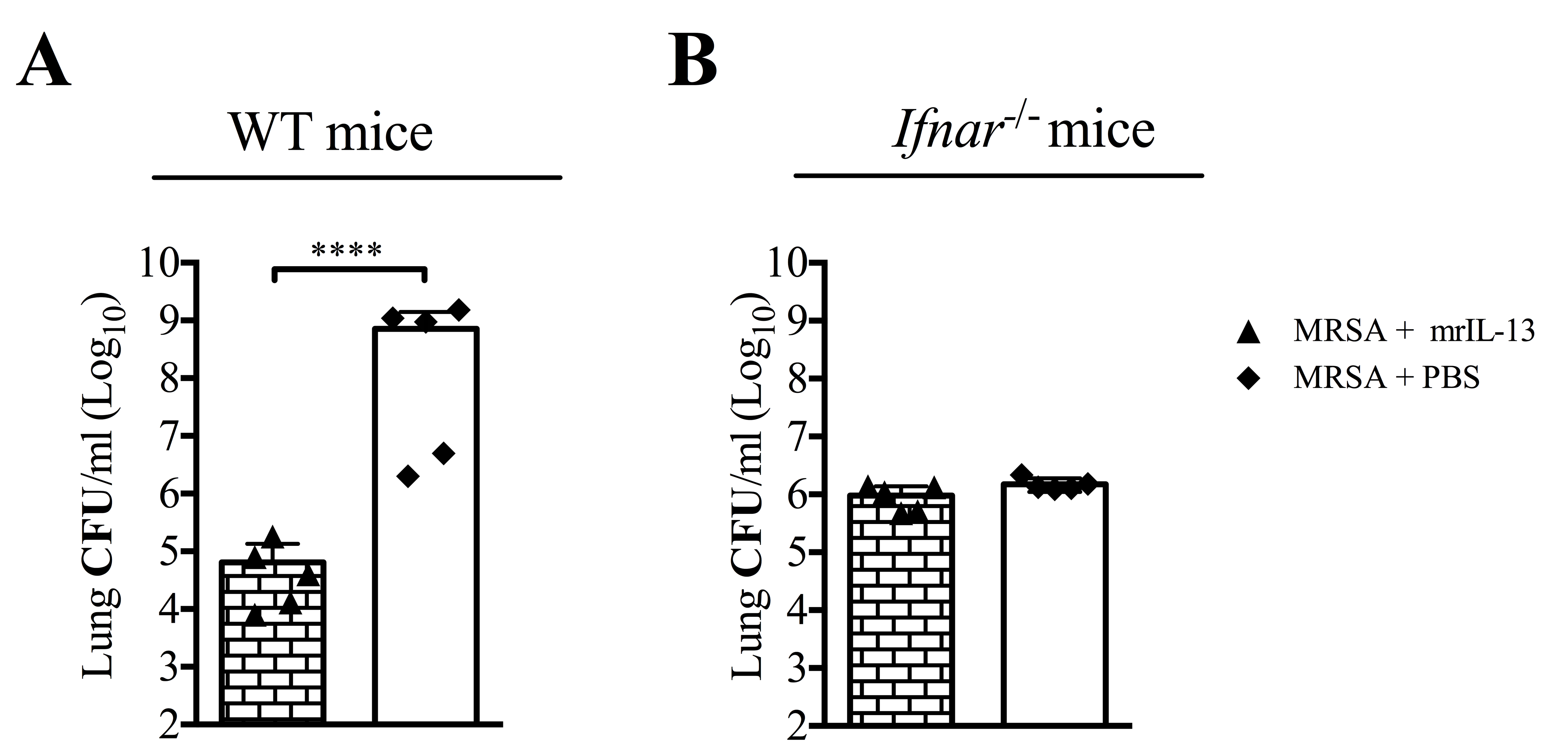

Supplement: Figure S1 — (A) C57BL/6 (WT) mice were infected with MRSA (3 × 10^8^ CFU). At the time of MRSA challenge and at 3 and 6 h after MRSA infection, mice received i.t. instillations of mrIL-13 (1 µg/dose). (B) Ifnar1−/− mice were infected with MRSA (1 × 10^8^ CFU). At the time of MRSA challenge and at 3 and 6 h after MRSA infection, mice received i.t. instillations of mrIL-13 (0.5 µg/dose). Lung bacterial burdens were evaluated at 24 h after MRSA infection. ****, P < 0.0001. Download [file mbo002162798sf1.tif]

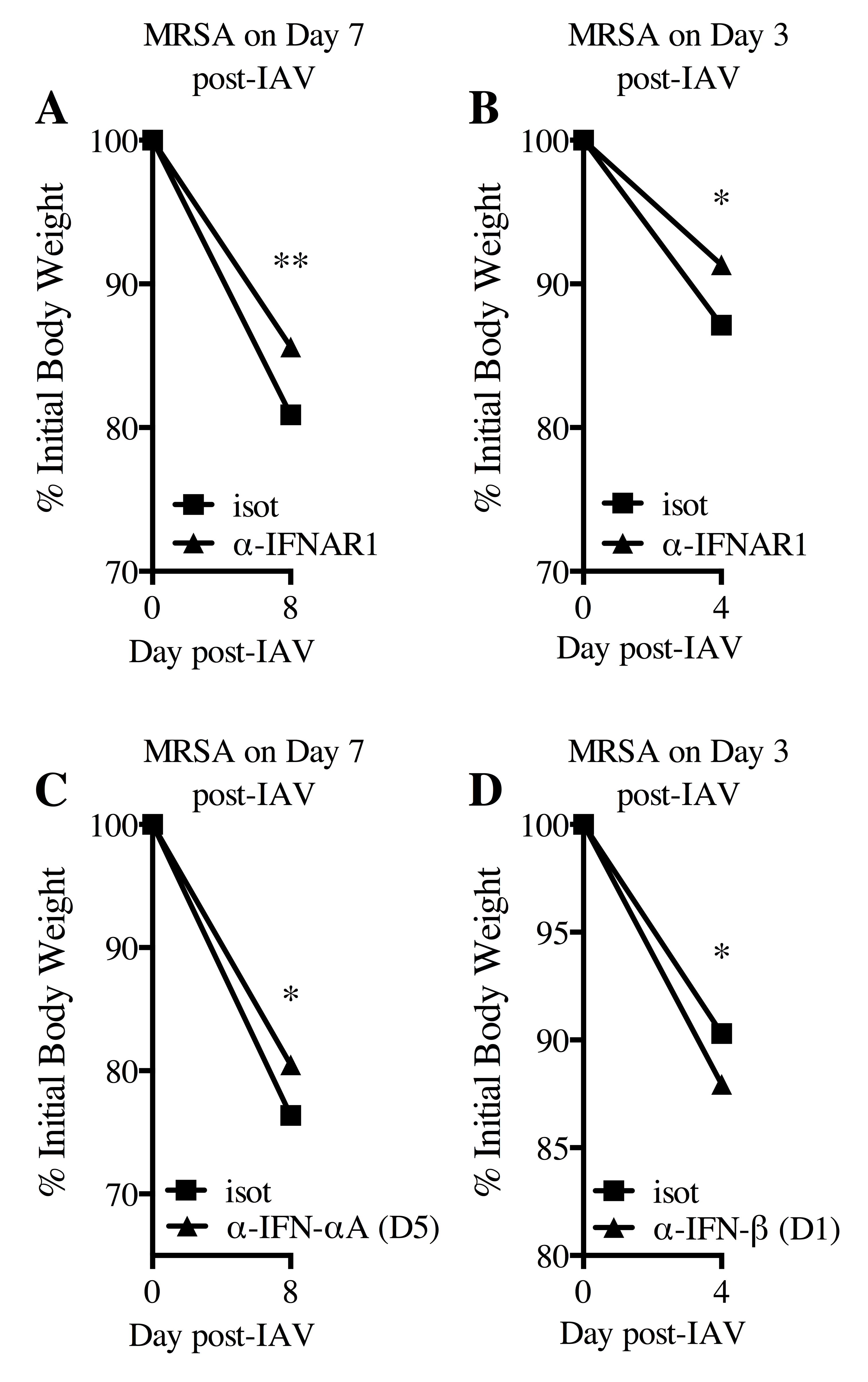

Supplement: Figure S2 — Body weight data from the experiments shown in Fig. 3. (A) WT mice were infected with IAV (day 0), treated with anti-IFNAR1 antibody on day 6, and challenged with MRSA on day 7. (B) WT mice were infected with IAV on day 0, treated with anti-IFNAR1 antibody on day 2, and challenged with MRSA on day 3. (C) WT mice were infected with IAV on day 0, treated with anti-IFN-αA or isotype antibody on day 5, and challenged with MRSA on day 7. (D) WT mice were infected with IAV on day 0, treated with anti-IFN-β or isotype antibody on day 1, and challenged with MRSA on day 3. **, P < 0.01; *, P < 0.05. Download [file mbo002162798sf2.tif]

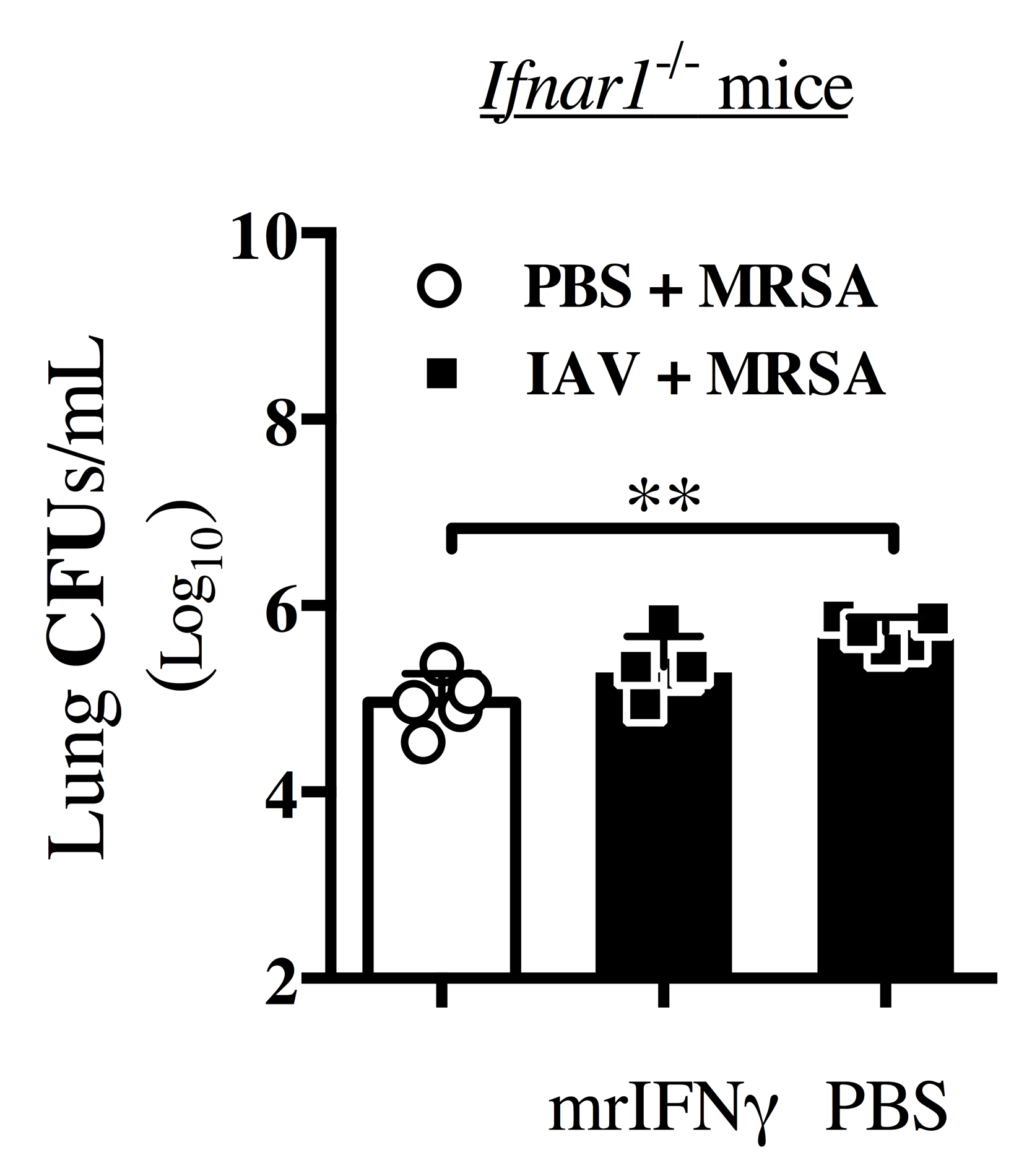

Supplement: Figure S3 — (A) IFN-γ−/− and C57BL/6 (WT) mice were infected with IAV on day 0 and challenged with MRSA on day 7. The levels of IFN-γ were evaluated in cell-free BALF collected at the time of sacrifice (day 8 post-IAV infection). (B) Ifnar1−/− mice were infected with IAV on day 0, treated with 1.5 µg mrIFN-γ on day 5, and challenged with MRSA on day 7. The lung bacterial burden was evaluated at 24 h after MRSA infection (day 8 post-IAV infection). **, P < 0.01. Download [file mbo002162798sf3.tif]

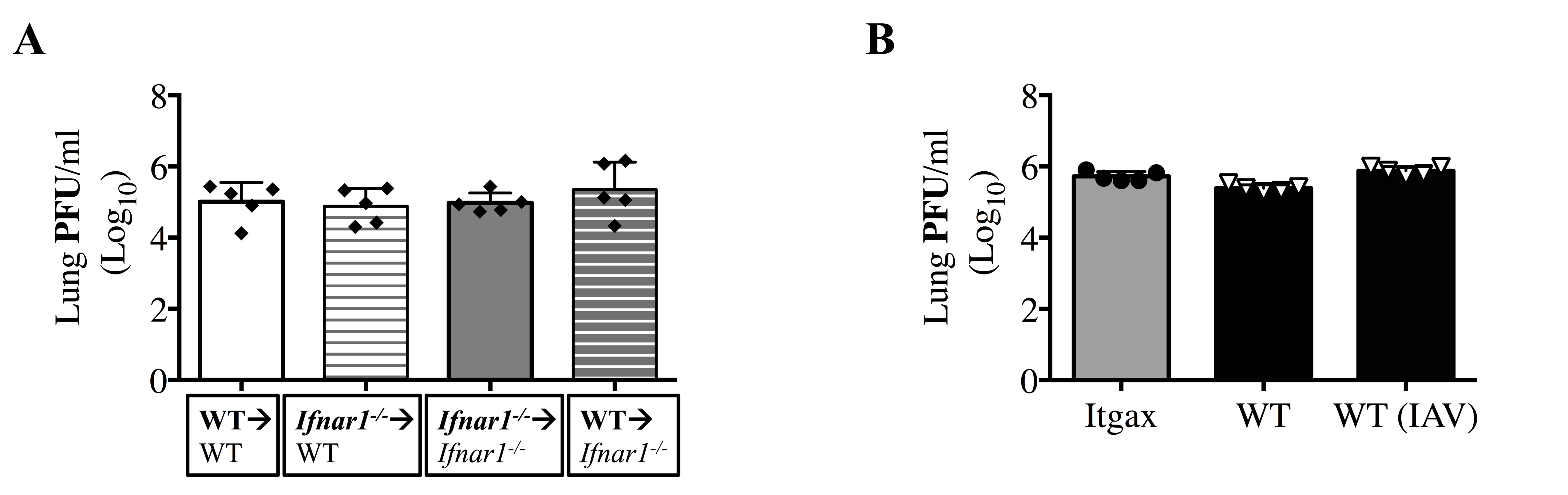

Supplement: Figure S4 — BM chimeric mice were infected with IAV on day 0 and challenged with MRSA 3 days later (the donor genotype is shown in bold, with an arrow indicating the recipient). (A) Viral burden was measured in the lungs 24 h after MRSA challenge (day 4 of IAV infection). Itgax (Ifnar1fl/fl;Itgax-cre) and WT mice were infected with IAV (or inoculated with PBS) and challenged with MRSA (or PBS) 3 days later. (F) The viral load was evaluated 24 h after MRSA challenge (day 4 post-IAV infection). Download [file mbo002162798sf4.tif]

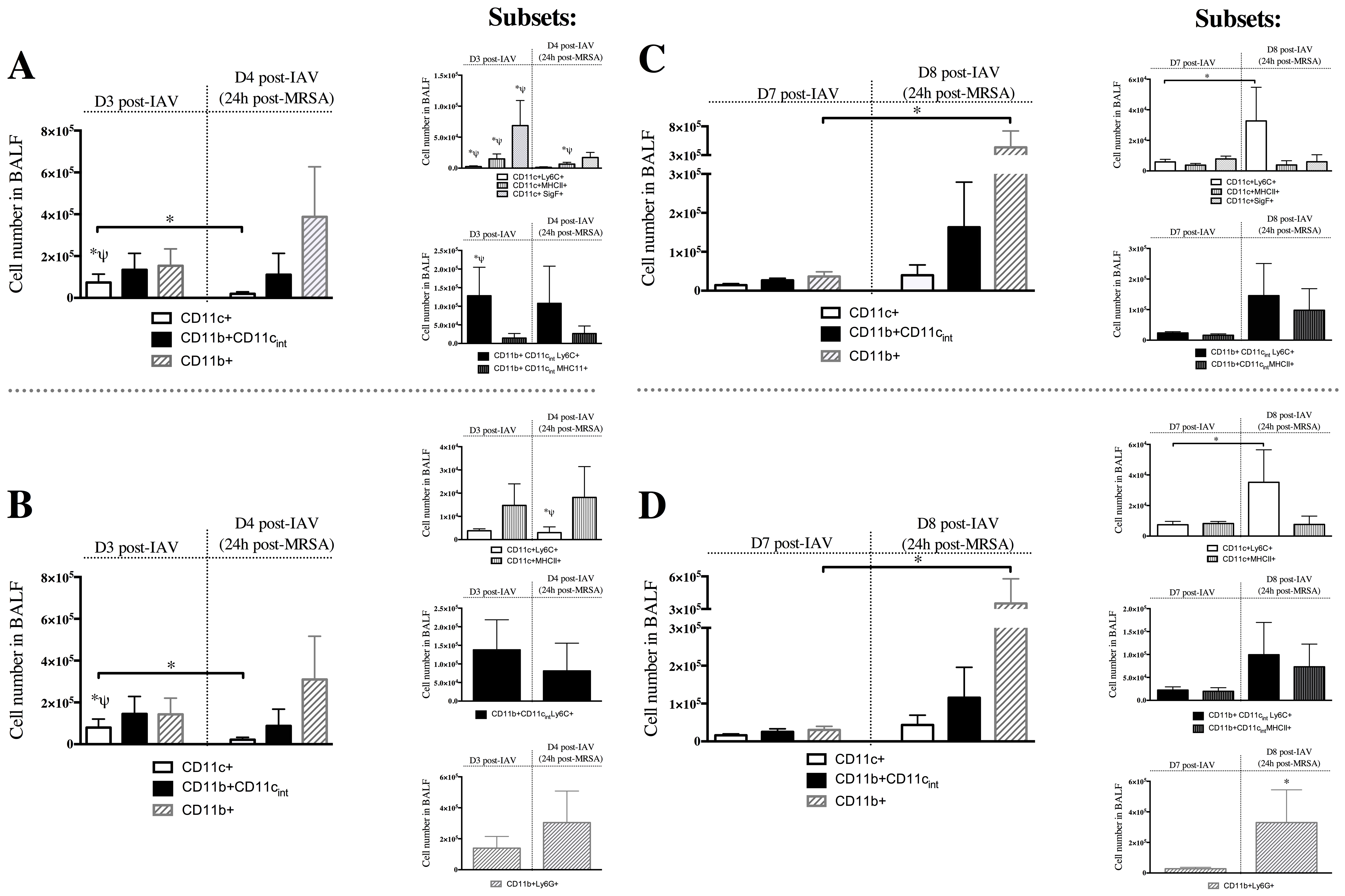

Supplement: Figure S5 — WT mice were sacrificed at the designated times post-IAV infection and post-MRSA infection (day 3 [d3], d4, and d7 or d8 post-IAV infection). Cells isolated from the BALF were stained and analyzed by FACS. The live cells gate was set on forward scatter (FCS) versus side scatter (SSC). Staining for CD11c versus CD11b was determined by gating total live cells. The gating strategy was as follows: for station 1 (A and C), major gates I (CD11c+ CD11b−), II (CD11b+ CD11cint), and III (CD11b+ CD11c−); subset gates I (SiglecF+, Ly6C+, and MHC-II+), II (Ly6C+ and MHC-II+); III (Ly6C−). For station 2, subset gates were as follows: I (Ly6C+ and MHC-II+), II (Ly6C+ and MHC-II+), and III (Ly6G+). The number of whole cells for major and subset gates are displayed. *, P < 0.05; ψ combined with *, the P value is for comparison of that cell type at day 3 and at day 7. Download [file mbo002162798sf5.tif]

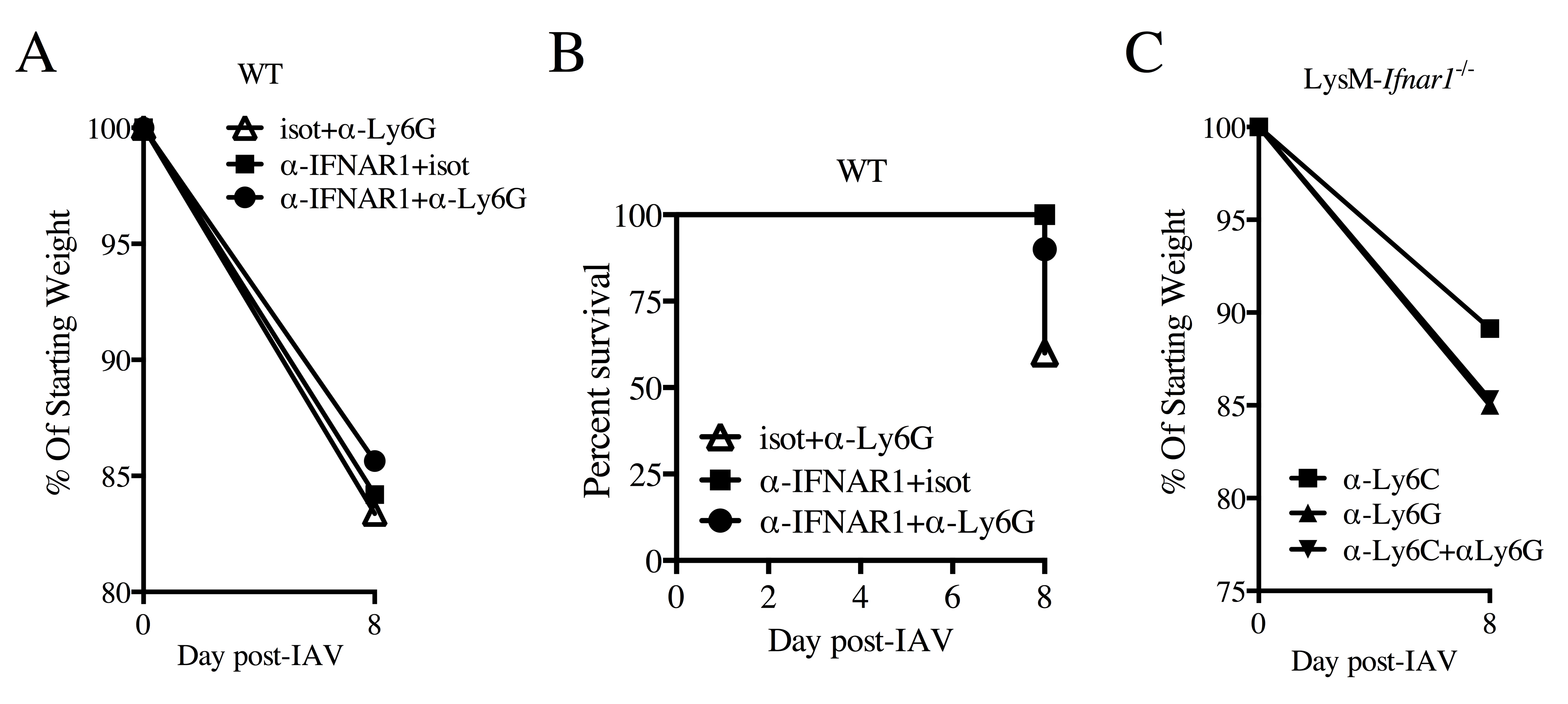

Supplement: Figure S6 — Body weight and survival data from Fig. 3 experiments. (A and B; extending data presented in Fig. 7B) WT mice were infected with IAV on day 0, treated with anti-IFNAR1 antibody on day 5.5 and/or anti-Ly6G antibody on day 6.5, and then infected with MRSA on day 7. (C; extending data presented in Fig. 7C) LysM-Ifnar mice were infected with IAV on day 0, treated with antibody (anti-Ly6G, anti-Ly6C, or both) on day 6.5, and infected with MRSA on day 7. Download [file mbo002162798sf6.tif]

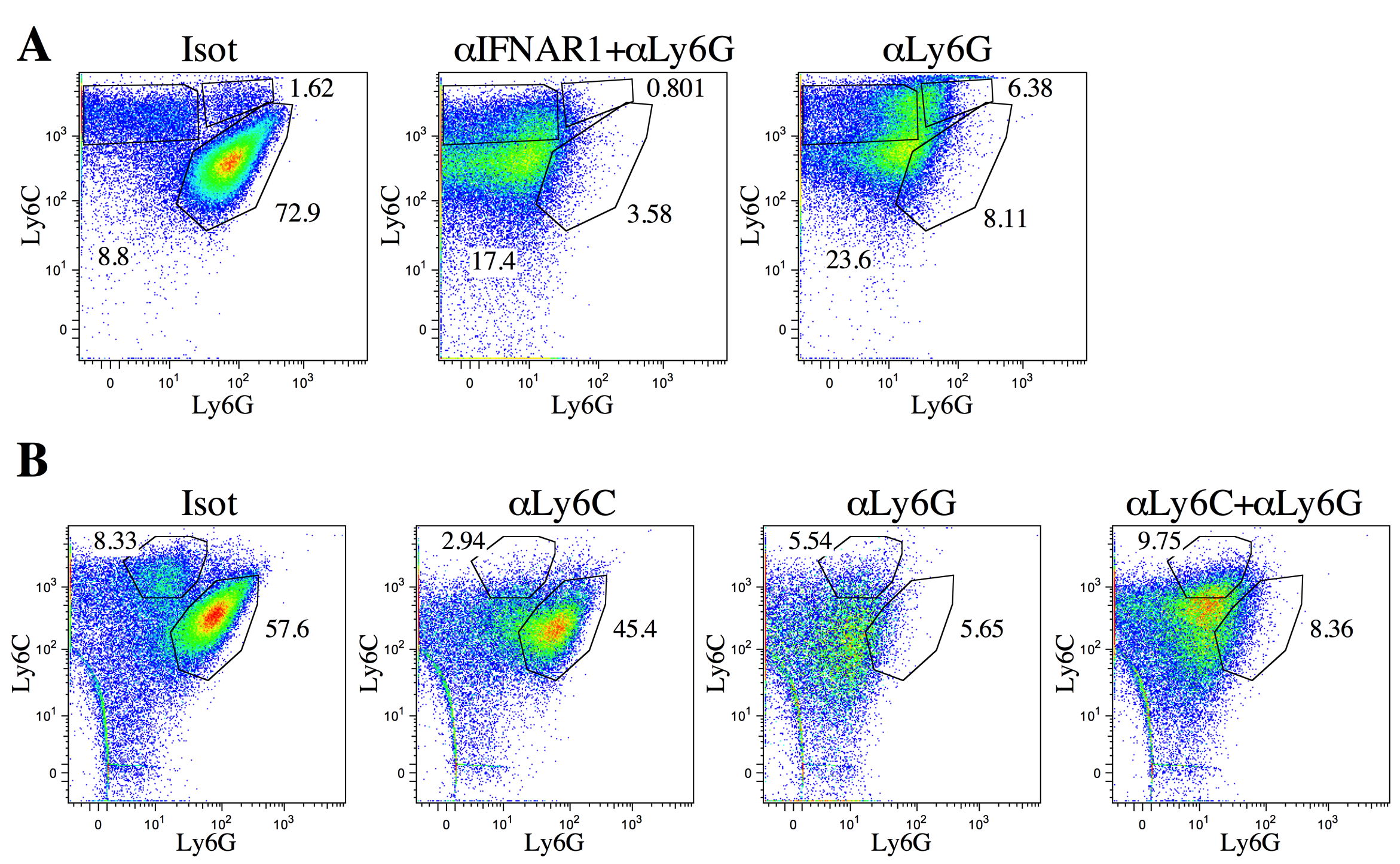

Supplement: Figure S7 — Cellular depletion plots for the data presented in Fig. 7B (A) and Fig. 7C (B). Cells isolated from the BALF were stained and analyzed by FACS. The live cells gate was set for forward scatter (FCS) versus side scatter (SSC). Staining for CD11c versus CD11b was determined by gating on total live cells. Plots shown are CD11b+ cells stained for Ly6G and Ly6C. (A) WT mice were infected with IAV on day 0, treated with anti-IFNAR1 antibody on day 5.5 and/or anti-Ly6G antibody on day 6.5, and then infected with MRSA on day 7. (B) LysM-Ifnar mice were infected with IAV on day 0, treated with antibody (anti-Ly6G, anti-Ly6C, or both) on day 6.5, and infected with MRSA on day 7. Download [file mbo002162798sf7.tif]
